# Deep learning for vessel segmentation and flow analysis to identify clusters associated with adverse outcomes in a fontan patient registry

**DOI:** 10.1038/s41598-026-40738-6

**Published:** 2026-03-04

**Authors:** Tina Yao, Nicole St. Clair, Madeline Gong, Gabriel F. Miller, Michael Quail, Shahin Moledina, Adam L. Dorfman, Mark A. Fogel, Rajesh Krishnamurthy, Christopher Z. Lam, Joshua D. Robinson, Timothy C. Slesnick, Justin Weigand, Jennifer A. Steeden, Rahul H. Rathod, Vivek Muthurangu

**Affiliations:** 1 Institute of Cardiovascular Science, University College London, London, UK; 2 Department of Cardiology, Boston Children’s Hospital, Boston, MA USA; 3 Congenital Heart Center, CS Mott Children’s Hospital, Ann Arbor, MI USA; 4 Division of Cardiology, The Children’s Hospital of Philadelphia, Philadelphia, PA USA; 5 Department of Radiology, Nationwide Children’s Hospital, Columbus, OH USA; 6 Department of Diagnostic Imaging, Hospital for Sick Children, Toronto, Canada; 7 Department of Pediatrics, Ann and Robert H Lurie Children’s Hospital of Chicago, Chicago, IL USA; 8 Department of Pediatric Cardiology, Emory University School of Medicine, Atlanta, GA USA; 9 Department of Cardiology, Texas Children’s Hospital, Houston, TX USA; 10 Department of Pediatrics, St. Louis Children’s Hospital, St. Louis, MO USA; 11 The Heart and Vascular Institute, UPMC Children’s Hospital of Pittsburgh, Pittsburgh, PA USA; 12 Department of Pediatric and Congenital Cardiology, Johns Hopkins Hospital, Baltimore, MD USA; 13 Division of Pediatric Cardiology, Seattle Children’s Hospital, Seattle, WA USA; 14 Division of Pediatric Cardiology, Rady Children’s Hospital, San Diego, CA USA; 15 Division of Pediatric Cardiology, University of Arizona, Tucson, AZ USA; 16 Division of Pediatric Cardiology, Riley Hospital for Children, Indianapolis, IN USA; 17 Division of Cardiology, Children’s National Hospital, Washington, DC USA; 18 Department of Bioengineering & Pediatrics, Stanford University, Palo Alto, CA USA; 19 Congenital Cardiology Unit, Ospedale Papa Giovanni XXIII, Bergamo, Italy; 20 Division of Pediatric Cardiology, University of Kentucky, Lexington, KY USA; 21 Division of Pediatric Cardiology, CHOC Children’s Hospital, Orange, CA USA; 22 Division of Pediatric Cardiology, Arkansas Children’s Hospital, Little Rock, AR USA; 23 Division of Pediatric Cardiology, Cedars-Sinai Guerin Children’s Hospital, Los Angeles, CA USA; 24 Division of Pediatric Cardiology, New York-Presbyterian Morgan Stanley Children’s Hospital, New York, NY USA; 25 Division of Pediatric Cardiology, Vanderbilt University Medical Center, Nashville, TN USA; 26 Division of Pediatric Cardiology, Yale New Haven Children’s Hospital, New Haven, CT USA; 27 Division of Pediatric Cardiology, Mount Sinai Kravis Children’s Hospital, New York, NY USA; 28 Division of Pediatric Cardiology, Stead Family Children’s Hospital, Iowa City, IA USA; 29 Division of Pediatric Cardiology, Oklahoma Children’s Hospital, Oklahoma City, OK USA

**Keywords:** Cardiology, Computational biology and bioinformatics, Diseases, Medical research

## Abstract

We introduce a deep learning framework comprising two models for automated segmentation (DCS) and large-scale deep temporal clustering (DTC) within a registry of single ventricle patients. The DCS model performs simultaneous classification and segmentation of velocity-encoded phase-contrast magnetic resonance (PCMR) data for five individual blood vessels, the left and right pulmonary arteries, aorta, superior vena cava, and inferior vena cava. Trained, validated and tested on 260 cardiac MRI exams (each containing 5 PCMR scans), it demonstrated a median Dice score of 0.91 on 50 unseen test exams. Integrated into a fully automated pipeline, the DCS model processed over 4500 registry exams without manual intervention, reaching 98% classification accuracy and 90% segmentation accuracy in cases with all five vessels present. Flow curves obtained from successful segmentations were used to train the DTC model, which performs deep temporal clustering to uncover unique flow patterns. Survival analysis showed that these groups were statistically correlated to increased risk of mortality or transplantation and to liver disease, highlighting the clinical relevance of the proposed framework.

**Supplementary Information:**

The online version contains supplementary material available at 10.1038/s41598-026-40738-6.

## Introduction

Time-varying signals can provide important insights into cardiac pathophysiology, capturing dynamic processes that static measurements don’t fully characterize. For instance, it is well-recognized that certain blood flow patterns are associated with specific disease processes (e.g. abnormal early/atrial filling ratio in diastolic dysfunction)^1^. One of the most accurate methods of measuring time-varying blood flow is velocity-encoded phase-contrast magnetic resonance imaging (PCMR), a technique that is heavily used in the evaluation of congenital heart disease (CHD)^2–9^. However, usually only time-averaged PCMR metrics (e.g. net forward volume) are reported, neglecting much of the information encoded into the time-varying signal. More sophisticated methods of leveraging the time-varying nature of PCMR data have been developed, but access to large amounts of data is required to validate their clinical relevance.

A potential source of large amounts of PCMR data is the Fontan Outcomes Registry using CMR Examinations (FORCE, http://www.forceregistry.org)^10^, which contains > 4500 cardiovascular MR exams performed in patients with functionally single ventricles and the Fontan circulation. These patients are of particular interest in our study because they are characterized by highly abnormal flow patterns^11–14^. The registry does contain PCMR images, but they are unprocessed and only time-averaged metrics like net forward volume are stored. Thus, investigation of time-varying flow requires the PCMR images to be reprocessed (segmented). However, manual segmentation would be too time-consuming, as well as being prone to inter- and intra-observer variability^15^.

Deep learning (DL) offers the possibility of automatically processing PCMR images, but there are two significant challenges specific to our application: (i) Images in the FORCE registry are heterogeneous due to differing CMR protocols/hardware at the contributing sites (common to all registries) and highly variable and complex anatomy (specific to Fontan patients), and (ii) There are multiple distinct vessels that must be first identified and then segmented if processing is to be performed without human input. Our solution is a joint Deep Classification + Segmentation model (DCS) that uses a modified UNet3 + architecture (that we have shown works with other images in the FORCE registry^16^) to simultaneously classify and segment multiple vessels.

Once large amounts of PCMR images are processed, novel methods for analyzing time-varying flow data (e.g. temporal clustering) can be developed and validated. Clustering attempts to group patients based on similar characteristics in some N-dimensional space and has been used to discover new phenotypes in several cardiovascular diseases^17–19^. Although conventional temporal clustering (e.g. dynamic time warping) has been used to analyze time-varying signals^20–23^, Deep Temporal Clustering (DTC)^24^ has been shown to have superior performance.

The overall aim of our study is to combine DL-based classification/segmentation with DL-based temporal clustering to investigate the time-varying blood flow in the Fontan circulation. The specific aims were: (i) Develop and validate a DCS model for classification and segmentation of five different phase-contrast flow planes: aorta (Ao), superior vena cava (SVC), inferior vena cava (IVC), left pulmonary artery (LPA) and right pulmonary artery (RPA), (ii) Integrate the DCS model into an automated pipeline to process the whole of the FORCE registry data (> 4500 datasets) and evaluate segmentation quality, (iii) Perform Deep Temporal Clustering (DTC) on extracted time-varying flow curves from the FORCE registry to identify novel flow-based phenotypes, and iv) Perform time-to-event analysis to assess the association of these phenotypes with key clinical outcomes, including death/transplantation and liver disease.

## Results

### Study overview

**Fig. 1:**
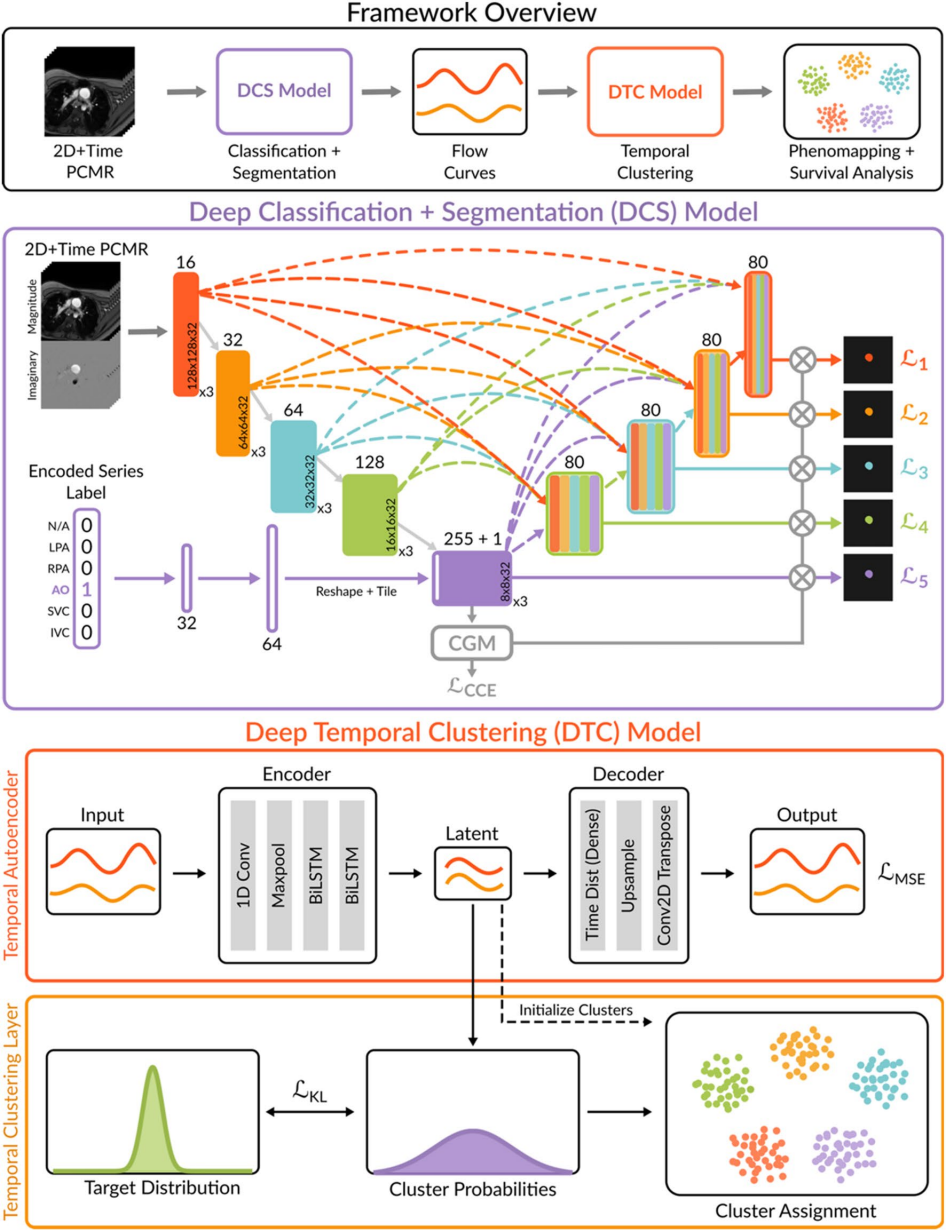
Overview of the deep learning framework for multi-vessel classification, segmentation, and phenomapping of phase-contrast MRI. The framework includes the Deep Classification + Segmentation (DCS) and Deep Temporal Clustering (DTC) models. DCS model: a 2D + time tunable UNet3 + with multi-class classification guidance. DTC model: a deep temporal clustering model architecture. MSE = Mean squared error, KL = Kullback–Leibler divergence, CGM = Classification-Guided Module adapted from UNet3 + ^32^.

Figure 1 shows the pipeline consisting of two DL models (full details in methods section), one for deep classification/segmentation (DCS) and one for deep temporal clustering (DTC). The DCS model was a multi-class UNet3 + architecture with full-scale skip connections, deep supervision, and a novel tunable input based on the DICOM series description. The model was trained, validated and tested on 260 manually segmented exams from the FORCE registry (training/validation/testing split into 185/25/50 exams, each exam contains five 2D + time PCMR series, data demographics are in Supplementary Table 1). The DCS model was then integrated into a fully automated processing pipeline, with segmentation results rated by an expert. These generated flow curves were then inputted into the DTC model. The DTC model was based on temporal autoencoder backbone that generated a latent space representation that was further optimized for clustering by minimizing the Kullback–Leibler (KL) divergence. Finally, flow-curve based clusters were associated with clinical outcomes (death/transplantation and liver disease).

### Deep classification + segmentation model

#### Classification performance

The DCS model achieved an overall classification accuracy of 97% across all vessels in the 50 unseen test exams (250 PCMR series). Classification accuracy for the Ao, SVC and IVC was 100%, and for LPA and RPA was 94% (Supplementary Fig. 1A).

The added value of the tunable layer that leverages series description information (entered by the technologist at the time of scanning) was tested by training a vanilla model without the tunable layer. This model achieved 94% accuracy across all vessels (compared to 97% with the tunable layer). The robustness of the tunable layer was evaluated by modifying the tunable layer input at inference in 2 ways: (i) removing series descriptions, which resulted in 94% accuracy; and (ii) assigning incorrect series descriptions in all cases, resulting in 90% accuracy.

Full confusion matrix results are shown in Supplementary Fig. 1, suggesting that the tunable layer increases classification accuracy without making the DCS model overly sensitive to erroneous or missing data.

#### Segmentation performance

Median Dice score across all test vessels (N = 250, Supplementary Fig. 2) was 0.91 (IQR: 0.86–0.93). Vessel-specific Dice scores were: Ao - 0.93 (IQR: 0.91–0.95), IVC - 0.93 (IQR: 0.89–0.95), SVC - 0.89 (IQR: 0.85–0.91), LPA - 0.90 (IQR: 0.87–0.93), and RPA - 0.88 (IQR: 0.84–0.92). The Ao and IVC Dice scores were statistically higher than other vessels (p < 0.007).

**Fig. 2:**
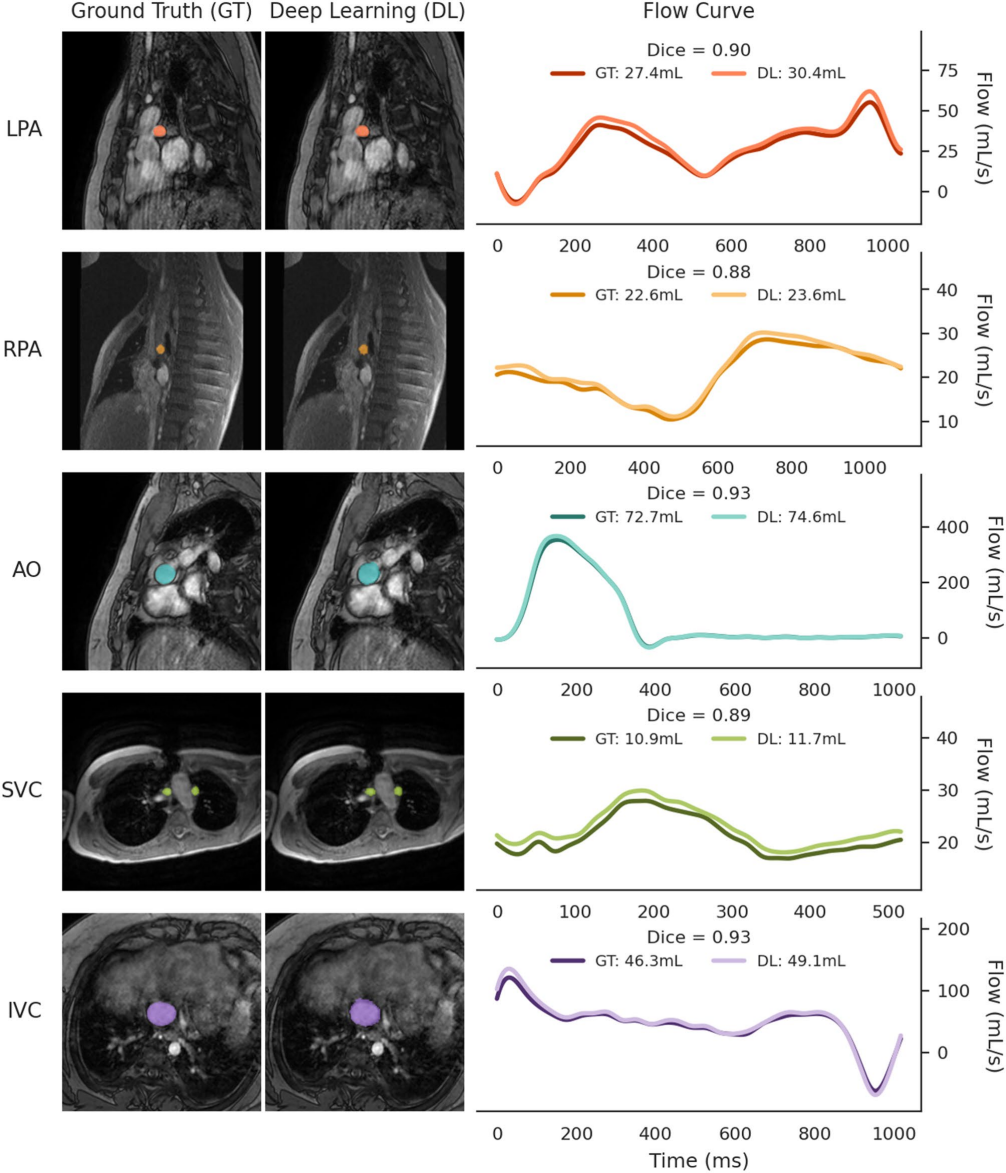
Comparison of ground truth and deep learning segmentations for each vessel. Dice scores were computed across the test cohort, and the case with the median Dice score is shown. Only the first frame of the image is displayed, along with the corresponding flow curves over the full cardiac cycle. Total forward volume over the cycle is reported in mL. Vessel labels: LPA – Left Pulmonary Artery, RPA – Right Pulmonary Artery, AO – Aorta, SVC – Superior Vena Cava, IVC – Inferior Vena Cava.

**Fig. 3:**
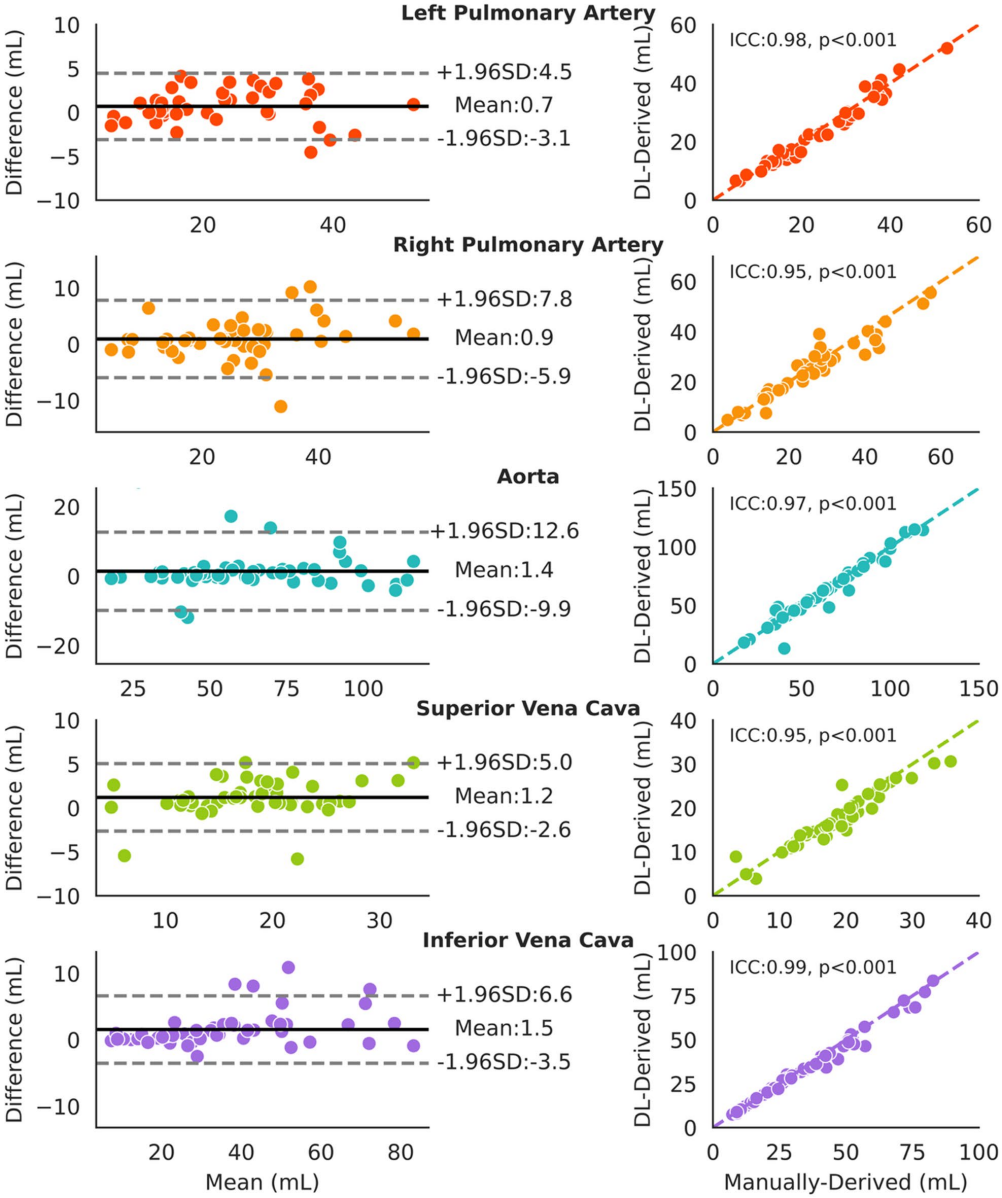
Comparison of total volume derived from deep learning and ground truth segmentations. Bland–Altman and correlation plots comparing total volume calculated from deep learning segmentations to ground truth segmentations across 50 test cases for each vessel.

Time-varying flow curves calculated from manual and DL segmentation are shown in Fig. 2 demonstrating good agreement over time for all vessels (further segmentation examples for each vessel in Supplementary Figs. 3–7). Comparison of net forward volumes calculated from these curves is shown in Fig. 3 with clinically acceptable limits of agreement and strong intraclass correlations (ICC > 0.95). However, DL segmentation did produce slightly higher net forward volumes than manual segmentations (2.2–6.7%), which were significant for the RPA, LPA, IVC, and SVC and trended for the Ao (p = 0.06).

The correlation between Dice accuracy and percentage accuracy of flow compared to the ground truth is shown in Supplementary Fig. 8. It shows that the better the segmentation, the closer the derived flow is to the ground truth flow.

#### Pipeline performance

At the time of the study, the FORCE registry contained 4881 CMR exams (3369 patients) with at least one PCMR series. Registry demographics are reported in Supplementary Table 2, with a median age at time of scanning being 15.8 (IQR: 11.3–21.9) years, similar proportions of patients with extracardiac and lateral tunnels, and the most common underlying diagnosis being hypoplastic left heart syndrome (HLHS).

The DCS model was integrated into a cloud-based automated pipeline (8 vCPUs, 120 GB RAM) that processed the whole registry (excluding the 260 exams used for model development). The processing time was 127 ± 80 s per exam, which included: (i) Sorting through all series in an exam (~ 31 series per exam), (ii) Extracting the PCMR series (~ 11 series per exam, noting that FORCE registry exams can include additional flow planes beyond the five the model was trained on, such as pulmonary veins or repeat scans), (iii) Running the joint model on all PCMR series (2.9s per series on the cloud instance, compared 218ms when run locally on a NVIDIA RTX A6000 GPU), (iv) Storing flow curves and segmentation masks for the LPA, RPA, Ao, SVC and IVC, and (v) Creating a GIF showing segmentation results for quality assurance. The total time to process the whole registry was ~ 170 h, with no human interaction required during this time.

Out of the 4881 exams, 2902 exams had PCMR data for all five vessels. In these exams, acceptable classification/segmentation (as assessed by a human expert, see methods) was achieved in 90% of all vessels. Individual rates were: LPA – 91%, RPA – 82%, Ao – 83%, SVC – 96%, IVC – 97% (Supplementary Fig. 9). Segmentation success was significantly higher in the SVC and IVC than in the LPA, RPA, and Ao (p < 0.001), while the LPA showed better segmentation success than the RPA and Ao (p < 0.001).

Failures were mainly due to inaccurate segmentation, with vessel misclassification only occurring in the LPA (~ 50% of failures) and RPA (~ 21% of failures). Failures were associated with poor image quality (p < 0.03 for the RPA and SVC) and certain anatomical/morphological features (Table 1). These included: (i) Similarly sized neo- and native aortas (bilateral aortas), which resulted in 51% successful aortic segmentations compared to 87% in the rest of the population (Supplementary Fig. 10 for example), (ii) Bilateral SVCs with only 90% successful SVC segmentations compared to 97% in single SVCs (Supplementary Fig. 10 shows a bilateral SVC failure), and also significant effects on segmentation success for the LPA (87% vs 92%) and RPA (75% vs 83%), and (iii) Heterotaxy, which affected segmentation success across all vessels except the Ao, particularly in the LPA (71% vs 94% in non-heterotaxy) and RPA (59% vs 85% in non-heterotaxy).

**Table 1:** Percentage of successful vessel segmentations for each vessel evaluated in the FORCE registry pipeline (N = 2902). Results are stratified by clinical and imaging variables. Values in bold denote statistical significance.

	**Overall**	**LPA**	**RPA**	**AO**	**SVC**	**IVC**
Overall	90	91	82	83	96	97
Age						
Child	90	91	82	82	97	98
Adult	90	93	83	83	96	97
*p*	0.602	**0.031**	0.872	0.409	0.502	0.436
Sex						
Male	90	92	83	83	96	97
Female	89	91	81	82	96	97
*p*	0.114	0.578	0.281	0.576	0.937	0.474
Image Quality						
Good	90	92	84	83	97	97
Poor	87	90	73	82	94	95
*P*	** < 0.001**	0.110	** < 0.001**	0.829	**0.027**	0.101
Bilateral Aorta						
No	91	91	81	87	97	97
Yes	84	93	86	51	93	98
*p*	** < 0.001**	**0.015**	**0.047**	** < 0.001**	**0.002**	0.249
Bilateral SVC						
No	90	92	83	82	97	97
Yes	86	87	75	85	90	95
*p*	** < 0.001**	**0.004**	** < 0.001**	0.227	** < 0.001**	0.173
Fontan Surgery						
Extracardiac	90	89	82	85	97	97
Lateral	90	94	83	80	96	98
Other	89	93	83	84	92	92
*p*	** < 0.001**	**0.004**	0.101	**0.031**	**0.004**	** < 0.001**
Heterotaxy						
No	91	94	85	82	97	98
Yes	80	71	59	84	93	95
*p*	** < 0.001**	** < 0.001**	** < 0.001**	0.492	**0.002**	**0.006**

p-values are from χ^2^ tests comparing groups.

LPA – Left Pulmonary Artery, RPA – Right Pulmonary Artery, AO – Aorta, SVC – Superior Vena Cava, IVC – Inferior Vena Cava.

### Deep temporal clustering model

Separate deep temporal clustering models were trained for the combined LPA/RPA flow curves (DTC_PA_), and the combined SVC/IVC flow curves (DTC_VC_). For both models, exams were only included if vessel segmentations were rated acceptable. Furthermore, aortic segmentations had to be rated as acceptable as aortic flow was used to identify and remove exams that were pulse-gated. This resulted in 1943 LPA/RPA flow curves and 2286 SVC/IVC flow curves being included in the clustering models.

#### Clustering

The optimal number of clusters, *k*, was determined using a sensitivity analysis with values from 3–8 (Supplementary Table 3). The DTC_PA_ and DTC_VC_ models did not converge with more than six and four clusters respectively. For values of *k* that did produce stable clusters, the optimal *k* was based on maximizing the temporal silhouette score and demonstrating significant differences for death/transplantation and liver outcomes where priority was given to death/transplantation. Based on these criteria, the optimum number of clusters was *k* = 5 for DTC_PA_ (silhouette score = 0.89, significant association with death/transplantation) and *k* = 4 for DTC_VC_ (silhouette score = 0.90, significant association with death/transplantation and liver disease).

Supplementary Fig. 11 presents t-distributed stochastic neighbor embedding (t-SNE) plots of the clusters before and after joint optimization with the clustering layer that enforces more confident predictions, demonstrating that the DTC method generates more distinct clusters compared to simple *k*-means on the latent space, which is similar to Principal Component Analysis (PCA)-based methods^25^.

##### Pulmonary artery (PA) clusters

**Fig. 4:**
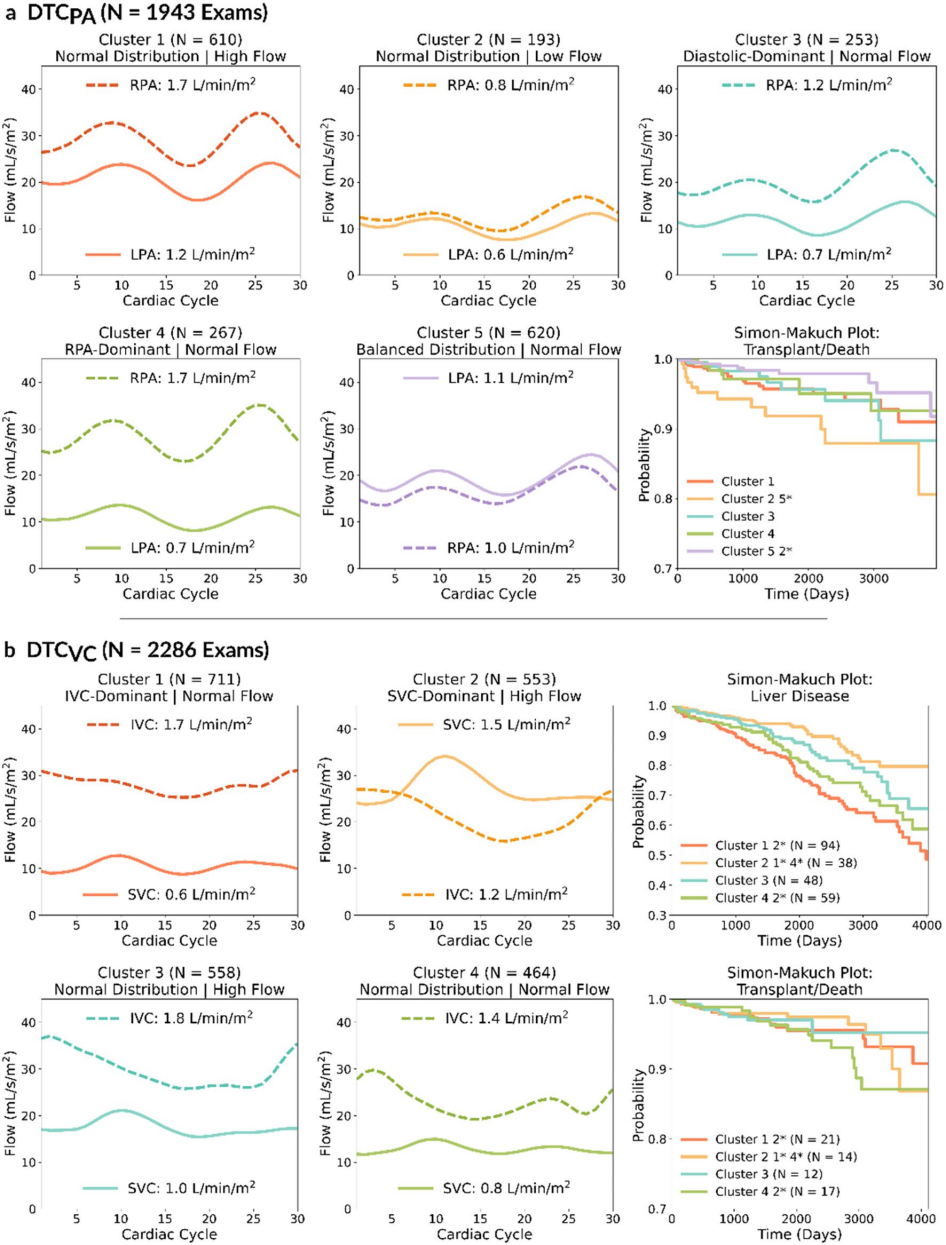
Mean centroid flow curves for each cluster alongside the mean flow for each vessel for both DTC models. The Simon-Makuch survival plot shows risk probabilities for liver disease and death/transplantation. (Liver disease is excluded for DTC_PA_ due to no significant group differences). The shaded regions indicate the confidence interval. LPA = Left Pulmonary Artery, RPA = Right Pulmonary Artery, SVC = Superior Vena Cava, IVC = Inferior Vena Cava. * Marks significant cluster differences according to time-varying Cox Regression.

Fig. 4A shows the mean flow curves for patients in each of the five PA clusters and Table 2 shows the key differences in patient demographics between the clusters (Full demographic information in Supplementary Table 4 and 5). The PA cluster characteristics are summarized as follows:

1. **Normal Distribution, High Flow (PA**_**Norm-High**_**):** This group had normally distributed branch PA flow, with higher flow towards the right pulmonary artery (42% LPA / 58% RPA) and high total pulmonary blood flow (2.91 L/min/m^2^). This was the youngest group (median age 13.7 years, p < 0.001 vs. all except PA_RPA-Norm_) and was 65% male. This group had EDV_i_ of 101.5 mL/m^2^, ESV_i_ of 47.5 mL/m^2^ and EF of 53%. The patients in this cluster also had the highest aortic flow (3.5 L/min/m^2^, p < 0.001) and lowest aorto-pulmonary collateral flow (18%, p < 0.04) compared to the other clusters. Fontan types were evenly split between lateral tunnel (46%) and extracardiac conduit (46%) and there was 6% heterotaxy.
2. **Normal Distribution, Low Flow (PA**_**Norm-Low**_**):** This group had normal distribution of branch PA flow (45% LPA / 55% RPA), but low overall total pulmonary blood flow (1.42 L/min/m^2^). This was the oldest group (median age 17.0 years, p < 0.002 vs. PA_Norm-High_ and PA_RPA-Norm_) and was 53% male. This group also had the highest ventricular volumes (EDV_i_ 105.6mL/m^2^, p < 0.001 vs. PA_Bal-Norm_; ESV_i_ 54.0 mL/m^2^, p < 0.002 vs. PA_Norm-High_ and PA_Bal-Norm_) and the lowest EF (49%, p < 0.03). In addition, they had the lowest aortic flow (2.5 L/min/m^2^, p < 0.001) and highest aorto-pulmonary collateral flow (25%, p < 0.001 vs. PA_Norm-High_ and PA_Bal-Norm_). Furthermore, this group had greatest proportion of extracardiac conduits (48%) as well as the highest prevalence of heterotaxy (12%, p < 0.008 vs. PA_Norm-High_ and PA_RPA-Norm_).
3. **Diastolic-Dominant, Normal Flow (PA**_**Dia-Norm**_**):** In this group, a greater proportion of branch PA flow occurred in during diastole, even though the total amount was normal (1.96 L/min/m^2^). This group was generally older (median age 16.6 years, p < 0.02 vs. PA_Norm-High_ and PA_RPA-Norm_) and 60% male. This group had EDV_i_ of 102.8mL/m^2^ and ESV_i_ of 51.3mL/m^2^, and EF of 51%. This group also had an aortic flow rate of 2.8L/min/m^2^ and a collateral flow of 23%. This group had 51% lateral tunnel, 43% extracardiac conduits and 7% heterotaxy.
4. **RPA-Dominant, Normal Flow (PA**_**RPA-Norm**_**):** In this group, flow was predominantly to the RPA (70%), with normal total flow (2.34 L/min/m^2^). This group was younger than average (median age 14.5 years, p < 0.002 vs. All expect PA_Norm-High_) and 65% were male. This group has EDV_i_ of 102.5mL/m^2^, ESV_i_ of 49.8mL/m^2^ and EF of 52%. They also had an aortic flow rate of 3.2 L/min/m^2^ and collateral flow of 23%. This group had 53% lateral tunnel and 43% extracardiac conduits and the least heterotaxy (3%, p < 0.001 vs. PA_Norm-Low_ and PA_Bal-Norm_).
5. **Balanced Distribution, Normal Flow (PA**_**Bal-Norm**_**):** This group had a balanced branch PA flow distribution where flow toward both pulmonary arteries were near-equal (52% LPA / 48% RPA) with normal total PA flow (2.18 L/min/m^2^). This group was older than the average (mean age 16.1) and 57% male. The ventricular volumes in this group were the lowest (EDV_i_ 94.5 mL/m^2^, p < 0.002 and ESV_i_ 44.4 mL/m^2^, p < 0.005), with an EF of 53%. This group had an aortic flow rate of 2.9 L/min/m^2^ and a collateral flow of 19%. This group also had the lowest proportion of lateral tunnels (38%) with a low proportion of extracardiac conduits (44%) and higher proportion of other Fontan types (18%). This group also had 11% heterotaxy.

**Table 2:** Demographics for the patients in the 5 identified clusters identified by DTC_PA_. Values﻿ in bold denote statistical significance.

	**All** **(N = 1943)**	**1: Normal distribution | High flow** **(N = 610)**	**2: Normal distribution | Low flow** **(N = 193)**	**3: Diastolic-dominant | Normal flow** **(N = 253)**	**4: RPA-dominant | Normal flow** **(N = 267)**	**5: Balanced distribution | Normal flow** **(N = 620)**	**p**
Total PA Flow	2.33 (1.92–2.73)	2.91 (2.59–3.28)	1.42 (1.21–1.67)	1.96 (1.72–2.12)	2.34 (2.16–2.59)	2.18 (1.88–2.45)	
Ratio of LPA Flow to Total PA Flow	0.43 (0.37–0.5)	0.42 (0.37–0.45)	0.45 (0.41–0.49)	0.38 (0.33–0.41)	0.3 (0.23–0.35)	0.52 (0.48–0.58)	** < 0.001**
Age	15.3 (11.1–20.8)	13.7 (10.4–18.4)	17.0 (11.4–23.3)	16.6 (12.6–22.5)	14.5 (10.8–19.3)	16.1 (12.0–22.8)	** < 0.001**
EDV_i_ (mL/m^2^)	99.2 (80.3–123.7) (N = 1915)	101.5 (84.2–124.0) (N = 595)	105.6 (82.3–135.5) (N = 192)	102.8 (82.3–125.9)	102.5 (84.8–130.8) (N = 265)	94.5 (76.1–115.4) (N = 610)	** < 0.001**
ESV_i_ (mL/m^2^)	47.5 (35.7–63.7) (N = 1911)	47.5 (36.5–62.2) (N = 594)	54.0 (39.5–73.0) (N = 191)	51.3 (37.5–66.8) (N = 251)	49.8 (35.9–67.2) (N = 265)	44.4 (32.5–58.7) (N = 610)	** < 0.001**
EF (%)	52 (45–57) (N = 1911)	53 (47–58) (N = 594)	49 (42–53) (N = 191)	51 (45–56) (N = 251)	52 (44–59) (N = 265)	53 (45–58) (N = 610)	** < 0.001**
Indexed AO Flow Rate (L/min/m^2^)	3.1 (2.6–3.6)	3.5 (3.0–4.0)	2.5 (2.1–3.1)	2.8 (2.4–3.3)	3.2 (2.7–3.7)	2.9 (2.5–3.3)	** < 0.001**
Collateral (%)	21 (12–32) (N = 1523)	18 (10–28) (N = 463)	25 (17–39) (N = 161)	23 (14–34) (N = 211)	23 (13–36) (N = 216)	19 (11–32) (N = 472)	** < 0.001**
Sex							
Male	1175 (60)	395 (65)	102 (53)	152 (60)	174 (65)	352 (57)	**0.004**
Female	768 (40)	215 (35)	91 (47)	101 (40)	93 (35)	268 (43)	
Fontan Type							** < 0.001**
Lateral	865 (45)	280 (46)	79 (41)	128 (51)	141 (53)	237 (38)	
Extracardiac	871 (45)	283 (46)	92 (48)	109 (43)	115 (43)	272 (44)	
Other Type	207 (11)	47 (8)	22 (11)	16 (6)	11 (4)	111 (18)	
Heterotaxy							** < 0.001**
No	1783 (92)	571 (94)	169 (88)	236 (93)	258 (97)	549 (89)	
Yes	151 (8)	36 (6)	23 (12)	17 (7)	8 (3)	67 (11)	

Continuous values are quoted as median (interquartile range) and categorical variables are number (percentage).

EDV_i_ – indexed end-diastolic volume, ESV_i_ – indexed end-systolic volume, EF – ejection fraction, LPA – Left Pulmonary Artery, RPA – Right Pulmonary Artery, AO – Aorta, SVC – Superior Vena Cava, IVC – Inferior Vena Cava.

##### Vena caval (VC) clusters

Fig. 4B shows the mean flow curves for patients in each of the four VC clusters and Table 3 shows key demographics differences between the clusters (Full demographic information in Supplementary Table 6 and 7). The characteristics of the VC clusters are summarized as follows:

1. **IVC-Dominant, Normal Flow (VC**_**IVC-Norm**_**):** This group had a higher proportion of flow from the IVC (72%) with an average amount of total vena caval flow (2.32 L/min/m^2^). This was the oldest group (median age 20.6 years, 66% adult, p < 0.001), and 59% were male. This group had EDV_i_ of 98.8mL/m^2^, ESV_i_ of 47.5mL/m^2^, and EF of 51%. This group had an aortic flow of 2.8L/min/m^2^ and collateral flow of 20%. This group also had the highest prevalence of lateral tunnel procedures (56%), lowest extracardiac conduits (32%) and 10% heterotaxy.
2. **SVC-Dominant, High Flow (VC**_**SVC-High**_**):** Greater proportion of flow from the SVC (56%) with high total vena caval flow of 2.83 L/min/m^2^. This was the youngest group (median age 8.2 years, 95% pediatric, p < 0.001), and 63% were male. This group had an EDV_i_ of 98.6mL/m^2^ and ESV_i_ of 46.7mL/m^2^, as well as the highest EF (53%, p = 0.001 vs. VC_Norm-Norm_). This group also had the highest aortic flow rate (3.7L/min/m^2^, p < 0.001) and one of the highest collateral flows (24%, p < 0.001 vs. VC_IVC-Norm_ and VC_Norm-High_). This group also had the highest prevalence of extracardiac conduit procedures (65%), lowest lateral tunnel (27%) and generally higher prevalence of heterotaxy compared to the other clusters (12%, p = 0.02 vs. VC_Norm-High_).
3. **Normal Distribution, High Flow (VC**_**Norm-High**_**):** Normal flow distribution (37% SVC / 63% IVC) with high total flow (2.86 L/min/m^2^). This group was predominantly younger (median age 13.5, p < 0.001) and 64% male. This group has EDV_i_ of 101.4mL/m^2^, ESV_i_ of 47.3mL/m^2^ and EF of 52%. This group had an average aortic flow rate of 3.3L/min/m^2^ and the lowest collateral flow (18%, p < 0.008). This group had 49% extracardiac conduits and lateral tunnel procedures of 43%, as well as the lowest prevalence of heterotaxy (7%, p < 0.02 vs. VC_SVC-High_ and VC_Norm-Norm_).
4. **Normal Distribution, Normal Flow (VC**_**Norm-Norm**_**):** Normal flow distribution (37% SVC / 63% IVC) with normal amount of flow (2.15 L/min/m^2^). This group was predominantly older (median age 17.2) and 57% male. This group had EDV_i_ of 99.7mL/m^2^ and ESV_i_ of 50.2mL/m^2^ and the lowest EF (51%, p < 0.02 vs. VC_SVC-High_ and VC_Norm-High_). They also had the lowest aortic flow rate (2.7L/min/m^2^ vs. all except VC_IVC-Norm_) and one of the highest collateral flows (24%, p < 0.002 vs. VC_IVC-Norm_ and VC_Norm-High_). This group had a similar amount of lateral tunnel and extracardiac conduits (44% and 43%) as well as the highest prevalence of heterotaxy (14%, p = 0.001 vs. VC_Norm-High_).

**Table 3:** Demographics for the patients in the 4 identified clusters identified by DTC_VC_. Values﻿ in bold denote statistical significance.

	**All** **(N = 2286)**	**1: IVC-dominant | Normal flow** **(N = 711)**	**2: SVC-dominant | High flow** **(N = 553)**	**3: Normal distribution | High flow** **(N = 558)**	**4: Normal distribution | Normal flow** **(N = 464)**	**p**
Total VC Flow	2.50 (2.10–2.98)	2.32 (2.02–2.63)	2.83 (2.42–3.33)	2.86 (2.44–3.25)	2.15 (1.75–2.52)	
Ratio of SVC Flow to Total VC Flow	0.36 (0.30–0.47)	0.28 (0.24–0.31)	0.56 (0.48–0.65)	0.37 (0.33–0.43)	0.37 (0.32–0.41)	** < 0.001**
Age	15.3 (10.8–20.6)	20.6 (16.7–26.5)	8.2 (5.4–10.8)	13.5 (11.3–16.4)	17.2 (14.3–22.5)	** < 0.001**
EDV_i_ (mL/m^2^)	99.5 (80.8–123.9) (N = 2262)	98.8 (79.7–123.2) (N = 708)	98.6 (79.9–123.6) (N = 548)	101.4 (81.9–122.0) (N = 544)	99.7 (83.4–127.6) (N = 462)	0.671
ESV_i_ (mL/m^2^)	47.7 (35.8–64.0) (N = 2257)	47.5 (34.8–64.9) (N = 708)	46.7 (35.7–62.1) (N = 545)	47.3 (36.3–62.2) (N = 542)	50.2 (36.7–68.0) (N = 462)	0.138
EF (%)	52 (45–57) (N = 2257)	51 (45–57) (N = 708)	53 (46–58) (N = 545)	52 (45–58) (N = 542)	51 (43–56) (N = 462)	**0.002**
Indexed AO Flow Rate (L/min/m^2^)	3.1 (2.6–3.7)	2.8 (2.4–3.2)	3.7 (3.1–4.3)	3.3 (2.9–3.7)	2.7 (2.3–3.2)	** < 0.001**
Collateral (%)	21 (11–33) (N = 1887)	20 (12–30) (N = 593)	24 (14–36) (N = 468)	18 (9–29) (N = 430)	24 (13–38) (N = 396)	** < 0.001**
Sex						
Male	1384 (61)	417 (59)	347 (63)	355 (64)	265 (57)	0.082
Female	902 (39)	294 (41)	206 (37)	203 (36)	199 (43)	
Fontan Type						** < 0.001**
Lateral	995 (44)	401 (56)	147 (27)	242 (43)	205 (44)	
Extracardiac	1056 (46)	226 (32)	358 (65)	274 (49)	198 (43)	
Other Type	235 (10)	84 (12)	48 (9)	42 (8)	61 (13)	
Heterotaxy						**0.01**
No	2033 (89)	633 (89)	485 (88)	516 (92)	399 (86)	
Yes	243 (11)	74 (10)	65 (12)	41 (7)	63 (14)	

Continuous values are quoted as median (interquartile range) and categorical variables are number (percentage).

EDV_i_ – indexed end-diastolic volume, ESV_i_ – indexed end-systolic volume, EF – ejection fraction, LPA – Left Pulmonary Artery, RPA – Right Pulmonary Artery, AO – Aorta, SVC – Superior Vena Cava, IVC – Inferior Vena Cava.

#### Cluster transition analysis

Supplementary Fig. 12 illustrates cluster transitions among patients with multiple scans, showing changes from the first to the last scan, as well as transitions between consecutive scans. In the PA model, 323 patients had multiple scans with 424 individual cluster transitions. Of these, 48% of patients remained in the same cluster between their first and last scan, while 52% remained in the same cluster between consecutive scans.

In the VC model, 390 patients had multiple scans, corresponding to 524 separate transitions. While 40% of patients remained in the same VC cluster from their first to last scan, 48% remained in the same cluster between consecutive scans.

These findings indicate that patients can develop distinct flow profiles over time. However, as patients with multiple scans represent a relatively small subset of the overall cohort, further analysis is required to draw conclusions regarding their outcome trajectories.

#### Cluster outcome analysis

Given that patients have multiple scans and their cluster membership can change over time, we performed time-varying time-to-event analysis (time-varying Cox regression^26^ adjusted for age, sex, indexed aortic flow rate, and ejection fraction EF, and Simon-Makuch plots, an extension of the Kaplan–Meier method for time-varying groups^27,28^). We investigated two outcomes (i) Death or transplantation as a composite outcome (death/transplantation), and (ii) The first diagnosis of Fontan-associated liver disease.

The Simon-Makuch plot for death/transplantation in the PA clusters is shown in Fig. 4A, with hazard ratios for pairwise cluster comparisons presented in Supplementary Fig. 13. The PA_Bal-Norm_ group had a significantly lower risk of death/transplantation compared to the PA_Norm-Low_ group (HR = 0.33, p = 0.007). There was no significant difference in liver disease observed among these clusters.

Figure 4B shows Simon-Makuch plots for death/transplantation and liver disease for the vena caval clusters, with pairwise hazard ratios in Supplementary Fig. 14. The patients in the VC_SVC-High_ cluster had a significantly lower risk of death/transplantation compared to the patients in the VC_IVC-Norm_ (HR = 0.34, p = 0.023) and VC_Norm-Norm_ clusters (HR = 0.31, p = 0.010). The VC_SVC-High_ cluster also had a significantly lower risk of liver disease compared to the VC_IVC-Norm_ (HR = 0.58, p = 0.027) and the VC_Norm-Norm_ clusters (HR = 0.56, p = 0.018).

#### Comparison to PCA-based clustering

We used PCA-based *k*-means clustering to generate clusters (Supplementary Fig. 15) with the same number of clusters as the DTC method (Fig. 4) for direct comparison. The figures show that centroid flow patterns from both methods were similar, suggesting that the DTC method for generating a latent space representation is robust and the flow patterns observed are replicable. Furthermore, the DTC method generates more distinct clusters, as shown by higher silhouette scores (PA: 0.89, VC: 0.90) compared to the PCA method (PA: 0.12, VC: 0.20).

More importantly, the two methods differ in their ability to create clusters with prognostic association. The DTC method finds clusters that have significant differences in death/transplantation for both PA and VC vessels (p = 0.007, p < 0.023, respectively). Conversely, the PCA-based clusters only just reach significance for PAs (p = 0.049) and are non-significant for VCs. Nevertheless, for liver disease, both the DTC and PCA methods find similar significant differences between VC clusters.

## Discussion

This is the first study, to our knowledge, to develop a deep learning model for the simultaneous classification and segmentation of multiple vessels (imaged using PCMR) and flow-based clustering of Fontan patients. The key findings were: (i) The joint deep classification and segmentation model (DCS) demonstrated high accuracy in classifying and segmenting major blood vessels, (ii) Incorporating the DCS model into an automated pipeline allowed rapid processing of the complete FORCE registry (> 4500 exams), providing robust flow measurement for about 90% of vessels, (iii) The deep temporal clustering (DTC) approach identified distinct flow dynamics between patient clusters, and (iv) These clusters showed significant associations with major clinical outcomes, including death or transplantation and liver disease. Our unified approach helps unlock the full potential of phase-contrast MR (PCMR) images stored in the FORCE registry by identifying novel physiologically distinct groups with prognostic significance. Importantly, our approach could be easily adapted to other CMR registries that contain PCMR images (e.g. The Indicator Cohort or PVDOMICS^29,30^).

### Deep learning PCMR segmentation

Manual core-lab segmentation of the PCMR images in the FORCE registry (> 4500 exams) would take ~ 2000 person-hours (~ 50 working weeks of non-stop segmentation). This is not tractable, which is why we developed a DL-based processing pipeline that processed the entire registry without human interaction in ~ 170 h (even when run on a cloud CPU). Importantly, we demonstrated high Dice scores compared to manual segmentation, as well as strong agreement between flow curves derived from manual and DL segmentations. This is despite the challenging and complex anatomy of Fontan patients^31^. We believe this is primarily due to the underlying UNet3 + architecture^32^, which allows better multi-scale feature fusion and provides a richer understanding of both fine details and global context. Although we have previously used a UNet3 + to successfully segment short-axis images in the FORCE registry^16^, extensive modifications were made to optimize the architecture for flow segmentation. One of the most important modifications was the use of a single network to perform both classification and segmentation. Previous studies that have aimed to segment multiple vessels used separate classifiers and vessel-specific networks, which adds complexity to training and inference, while limiting information sharing between tasks^33^. In contrast, our simpler joint approach leverages the UNet3 + architecture and also introduces a novel tunable input based on series descriptions. This input provides contextual information added by technologists during CMR scans without hard-coding, thereby improving classification accuracy while remaining robust to missing (~ 7% of all exams) or incorrect descriptions. The network was also modified to accept 2D + time data by using 3D convolutions. This enabled feature learning between consecutive frames and enforced temporal consistency. Finally, the imaginary component of the complex signal was also included as an additional input to our model, which leveraged velocity information to improve accuracy without having to manage the high levels of noise present in phase images^33^.

Our models demonstrated 90% success across the FORCE registry, which includes data from various scanners, clinical sites and complex anatomies, providing very large amounts of data for flow-based clustering. Nevertheless, there were certain situations in which the joint classification + segmentation model performed less well. This included specific anatomies; for instance, heterotaxy seemed to confuse the model because the LPA can look like the normal RPA plane and vice versa.

Although the aorta had the highest Dice score in the test set (0.93), it had one of the lowest segmentation success rates in the full pipeline (83%). This discrepancy cannot only be explained by differences in the amount of bilateral aorta cases, as they were similar in the pipeline cohort (12%) compared to the test set (10%). This suggests that the pipeline cohort likely included greater variability or combinations of variability that are difficult to quantify or were not explicitly analyzed. For example, another main difference is that the test set was drawn from hospitals used for model training, whereas the pipeline involved inference on data from multiple previously unseen centers with potentially different underlying imaging characteristics.

If these cases were underrepresented in the training data, future performance could improve by including more examples of uncommon anatomies or unusual imaging protocols.

### Temporal clustering analysis

Deriving clinically useful biomarkers from time-varying data requires temporal dimensionality reduction. This is often achieved using simple methods (such as reporting peak or mean values), and for PCMR data, usually only net forward volumes are reported^34,35^. However, these simplistic approaches do not extract all the potentially useful physiological information from flow data. Principal component analysis offers a more sophisticated approach by decomposing temporal flow curves into weighted principal components (PCs) that account for the majority of variance in the populations. This method has already been applied to flow in the Fontan circulation, where poorer outcomes were associated with certain flow patterns (e.g. diastolic dominant PA flow)^36–38^. Furthermore, it is possible to combine PCA with *k*-means clustering to perform temporal clustering^39–42^. In our study, we demonstrated that although PCA-based clustering produced similar clusters to DTC, the DTC method produced more distinct clusters (as shown by higher silhouette scores) with more significant associations with outcomes. The poorer performance of PCA-based clustering is potentially related to the PCA being constrained by the linear summation of orthogonal PCs, which may limit the expressivity of the method. Therefore, we opted to use DTC, a method that uses a temporal autoencoder to generate a latent space that can capture more complex temporal patterns compared to using PC decomposition. This method also employs a joint optimization step to maximize the certainty of cluster assignment. Importantly, as our DSC pipeline model provided >20× more data than previous studies on blood flow in Fontan patients^36–38^, providing a dataset large enough for deep learning to reliably learn complex, high-dimensional temporal patterns without overfitting.

Deep temporal clustering analysis of the FORCE dataset revealed distinct flow patterns that in some cases were associated with clinically relevant outcomes. Patients in the PA_Norm-Low_ group (normal distribution, low flow) had an increased risk of death. This might be unsurprising, but our analysis was corrected for indexed aortic flow rate and EF, suggesting that this was not simply the result of poor perfusion and cardiac function. Interestingly, this group did have the highest systemic to pulmonary collateral flow, and one possibility is that they have higher pulmonary vascular resistance, which would explain their higher mortality. It is also possible that low pulsatility and diastolic-dominance in these patients reflect an additional adverse physiology that contributes to higher mortality. A more surprising finding is that the PA_Bal-Norm_ (balanced distribution, normal flow) group had the best outcome. This group had slightly greater flow to the left lung, which could not be fully explained by heterotaxy (although heterotaxy was slightly overrepresented). An intriguing explanation for these findings is that Fontan anatomy with equal or slightly greater left lung flow is associated with better hemodynamics and thus, improved outcome. This could be investigated by leveraging DL-based anatomical segmentation and flow field estimation^43^ to investigate power loss and TCPC resistance^44^.

We also found that patients in the VC_Norm-Norm_ group (normal distribution and normal flow) have an increased risk of death/transplantation. Conversely, patients with dominant SVC flow were associated with a lower risk of death/transplantation and liver disease. These patients were younger, which is expected as SVC/IVC flow ratio reduces with age. Nevertheless, the association with outcome remained even after correction for age, suggesting that other mechanisms resulted in better prognosis in these patients. One possibility is that increased splanchnic circulation is associated with worse mortality. This idea is supported by the fact that the cluster with dominant IVC flow also had higher mortality, and a potential mechanism is increased liver disease in both these groups. Another possible explanation is that higher SVC flow leads to less flow collision between the vena caval flow streams, resulting in great hemodynamic efficiency. Further investigation of flow distribution is required to better understand the reason for these associations, which is achievable using the comprehensive data available in the FORCE registry.

We have demonstrated the potential of deep temporal clustering for the analysis of time-varying signals at scale. This method could easily be applied to multiple time-varying signals in the cardiovascular imaging space, including flow in other pathologies, myocardial strain, and ventricular/atrial volumetric curves. However, use in large registries does require a joint segmentation-clustering approach.

### Limitations

The main limitation of our study was that segmentation was not successful in all cases, meaning that human review for all outputs of the DCS model was necessary. However, this can be done in a matter of seconds per exam, which is feasible even when reviewing thousands of exams.

Another issue is that our tunable input relies on a curated data dictionary to extract tokens from series descriptions. This dictionary was developed using descriptions from the current FORCE registry, so it may not perform well with new datasets that may have different naming conventions. This could be remedied by using a large language model (LLM) to represent the series descriptions as an embedding that can be inputted into the MLP of the tunable layer in place of the current one-hot encoding, with end-to-end training of both the UNet3 + and LLM. However, we demonstrated that our model does have high classification accuracy, and an LLM may be an unnecessary complication.

Another limitation is that we cannot assess the bias of segmentation failures on the clustering results, as only successful segmentations were used during training and inference of the clustering model. Furthermore, although we measured the number of transitions that a patient has between different scans according to their cluster membership. These findings indicate that patients can develop distinct flow profiles over time. However, as patients with multiple scans represent a relatively small subset of the overall cohort, further analysis is required to draw conclusions regarding their outcome trajectories.

One known issue with PCMR is the background phase, particularly with older exams. We didn’t perform background phase correction because software methods that aim to fit parabolic planes to the image are highly sensitive to the amount/segmentation of static tissue. In clinical use, these methods are checked and discarded if they are clearly incorrect. However, our pipeline is designed not to require significant human input, and background phase correction would be difficult under these conditions. While the lack of background phase correction can result in the flow offsets, our DTC method has been trained to cluster based on the shape of the flow curve as well as absolute flow values. Thus, we believe that clustering is partially robust to phase offsets. Furthermore, it is recognized that background phase errors are more prevalent with older scanners. If clustering was heavily influenced by background phase, one might expect difference in scan date between clusters. However, Supplementary Tables 4–7 show there were no significant differences found in scan date for any PA clusters. It should be noted that VC_SVC-High_ cluster scans were acquired significantly earlier than the other groups. However, patients in this cluster were also significantly younger, which may explain why the scans were older rather than due to bias from background phase effects.

Finally, we were unable to assess the effect of different types of acquisition (e.g. free breathing, breath-hold, and real-time) and known artefacts (e.g. due to stents) on both segmentation accuracy and cluster assignment. This is because this data wasn’t available in the FORCE registry, but the high success rate in segmentation does suggest good overall generalizability. Furthermore, although the models were validated on heterogeneous data from multiple scanners, protocols, and sites, they have not been tested on patients beyond the single-ventricle population and may perform less reliably on more typical cardiac anatomy. Nevertheless, the methods developed in this study could be applied to other datasets.

### Conclusion

In this study, we introduce a unified framework for flow segmentation and clustering. Our deep classification segmentation (DCS) model processes multiple blood vessels using PCMR images from different sites, scanners, and types of single-ventricle physiology. We validated the DCS model on a large dataset (2902 exams, 14,510 phase-contrast series), achieving an average success rate of 90% across all five vessels. By leveraging the flow curve data, we used DTC to identify clusters and analyze curve characteristics, linking them to clinical outcomes. We believe our method can provide new insights into Fontan physiology and potentially provide new methods of treatment. In addition, our methodology could easily be modified for other large cardiovascular datasets that contain time-varying signals (e.g. PCMR, strain data, or even ECG data). In a clinical environment, we envisage an automated system, applied directly after scanning, which segments all five vessels, to provide a full flow profile, and classifies the patients into higher or lower risk, without human input.

## Methods

This was a multicenter study approved by the Institutional Review Boards or research ethics committees at each participating institution or via a reliance Institutional Review Board agreement with Boston Children’s Hospital. The study proposal and this manuscript were approved by the FORCE Data Governance and Publications Committee. The study involved no direct participation of the patients. The complete list of FORCE Investigator co-authors and affiliations is enumerated in the Authors’ Contribution section.

### Deep classification + segmentation model

#### Model architecture

The Deep Classification + Segmentation model (DCS), shown in Fig. 1, is based on a five-scale UNet3 + architecture with increasing filters at each scale (16, 32, 64, 128 and 255). An additional tunable layer (1 filter) is concatenated at the bottleneck, increasing the total number of filters at this scale to 256. The modifications implemented to enable simultaneous classification and segmentation of multiple vessels are described below.

##### Dual channel input

PCMR images consist of both magnitude (anatomical) and phase (velocity) data, although only magnitude images are conventionally used for DL segmentation. Phase images can also be included, but high noise in areas of very low signal (e.g. air) can have a detrimental effect on DL. Thus, we converted the magnitude and phase images to a complex representation and then extracted the imaginary component. In the imaginary image, flowing blood has a high signal, while both static tissue and air have near-zero signal (Supplementary Movie 1). By using the magnitude and imaginary data as inputs, we were able to leverage the blood flow signal to aid segmentation without having to contend with the high noise in the phase data.

##### Full-scale skip connections and deep supervision

Inspired by the UNet3 + , we incorporated full-scale skip connections and deep supervision into our UNet^32^. These modifications integrate coarse and fine-grained features at multiple scales, and we have previously shown that this architecture is well-suited to the segmentation of complex and heterogeneous anatomy^16^.

##### Incorporating time

The DCS architecture utilizes 3D convolutional kernels that can process the 2D + time PCMR cine images in one pass. These 3D convolutions enable information to be passed between frames, producing more consistent segmentation across time. It should be noted that no maxpooling is performed in the time dimension to prevent unwanted temporal compression.

##### Tunable series description input

The DICOM series description is entered by the technologist at the time of scanning and often includes information about the imaging plane. Inspired by the tunable UNet^45^, this information was injected into the DCS network to aid classification. The DICOM series description was first converted into a one-hot encoded six-element vector with one entry for each vessel (LPA, RPA, SVC, IVC, and Ao) and one for empty or other series descriptions. This was achieved using a data dictionary containing predefined terms commonly associated with the five vessels (Supplementary Table 8, Tunable Input Data Dictionary). The one-hot encoded series description was then processed through a multi-layered perceptron (MLP) that outputs a 64-element output vector that is reshaped to 8 × 8 and tiled to 8 × 8 × 32 to match the size of the UNet bottleneck feature map. This was then concatenated with the bottleneck features to yield an 8 × 8 × 32 × 256 tensor. By incorporating these features into the bottleneck, the model can leverage additional information about the imaging plane in the decoding arm to aid classification.

##### Multiclass classification

We incorporated a multichannel output in which each channel was dedicated to producing a specific vessel segmentation mask, enabling joint classification and segmentation. We augmented the multiclass classification by including a Classification-Guided Module (CGM), also inspired by the UNet3 + ^32^. The CGM was an additional branch from the bottleneck layer that attempted to constrain segmentations to a single output channel. The multiclass classification module in the DCS model generates a six-class classification output: one for each vessel and one for the background.

#### Training data

The training dataset included 260 CMR exams from single ventricle patients in the FORCE registry, comprising 1300 individual PCMR series (each exam contains one for each vessel: LPA, RPA, SVC, IVC, and Ao). Of these, 185 exams were used for training, 25 for validation, and 50 for testing. The training set maintained a similar site distribution to the FORCE registry database, while the validation and test datasets had approximately equal numbers from each site. Supplementary Table 1 illustrates the demographic information of the ground truth data. No significant differences in BSA, age, sex, situs type, heterotaxy, diagnosis, type of Fontan surgery, or dominant ventricle were found between the training, validation, and test datasets.

A clinical researcher (N.S.C.) with five years of cardiac imaging experience identified phase-contrast exams for each of the five vessels, segmenting only the vessel of interest for each view, even if other vessels were visible (e.g., the aorta in an SVC-specific plane). We specifically selected patient exams with all five vessels scanned to enable direct comparison across patients and vessels and because flow measurements from all vessels are required for the deep temporal clustering method. The vessels were contoured over the entire cardiac cycle using a semi-automatic algorithm with manual correction (Circle cvi42 version 6.1.2; Circle Cardiovascular Imaging).

#### Preprocessing, model training, and post-processing

Data preprocessing included the pixel size being reduced to 2 × 2mm spatial resolution using spline interpolation, and the resultant images being center-cropped or padded to a size of 128 × 128 pixels. Reducing the image size helped to keep memory requirements down during training and had no discernible effect on final accuracy. The number of time frames was interpolated to 32 (median for all ground truth exams) using spline interpolation. Contrast-limited adaptive histogram equalization (CLAHE) was applied to the magnitude images to improve contrast and generalizability^46^.

The DCS model was trained using a weighted combination of focal Tversky loss for segmentation and categorical cross-entropy for the classification-guided module, with respective weights of 1.00 and 0.25. The combined loss was calculated at each deep supervision layer, with the first four layers equally weighted at 0.25 and the final layer at 1.00. The model was trained for 400 epochs with a batch size of 8 using the Adam optimizer. Image augmentation included random on-the-fly variations in rotation, translation, brightness, contrast, cropping, padding, and resizing to improve the model’s segmentation robustness. The series description (represented as a one-hot encoded vector) was also randomly assigned in 5% of cases to help the model tolerate missing or incorrect labels. To ensure the model was invariant to the direction of flow encoding, the sign of the imaginary pixel values was alternatingly inverted during training.

During post-processing, only the largest connected components of the LPA, RPA, and IVC masks were retained. For the aortic and SVC masks, the two largest components were kept to account for neo and native aortas or bilateral SVCs. The predicted segmentation masks were then resized and projected onto the original PCMR images. Flow curves were generated from both DL and manual segmentation as previously described^47^, and net forward volumes were calculated by integrating the flow curve over time.

#### *Deep classification* + *segmentation model evaluation*

The Deep Classification + Segmentation (DCS) model was validated on five vessel planes from the 50 ground truth test datasets (250 PCMR series). The classification accuracy of our model was evaluated per vessel.

To evaluate the importance of the tunable layer, we also trained a vanilla DCS model without the series description input layer. Furthermore, we assessed the robustness of the tunable layer by additionally testing the model at inference with 2 different tunable layer inputs: (i) using missing series descriptions (one-hot encoding the ‘missing’ entry); and (ii) using incorrect series descriptions (randomly selecting the wrong vessel entry in one-hot encoding).

Segmentation accuracy of the DCS model was assessed using the Dice score between the predicted outputs and ground truth. For cases with Dice scores above zero, flow curves and net forward volumes derived from predicted and ground truth data were compared.

### Pipeline overview

We developed an automated pipeline to apply the DCS model to phase-contrast images in the FORCE registry without human input. The pipeline uses DICOM headers to identify phase-contrast series from CMR exams, runs the DCS model to classify and segment the image planes, and applies rule-based methods to select the most appropriate vessel plane when multiple series are classified as the same vessel by the model. Full details are reported in Supplementary Sect. “Pipeline Overview”.

The clinical researcher (N.S.C.), who manually segmented the ground truth data, reviewed all segmentations produced by the automated pipeline. For every exam, each vessel’s segmentation was individually rated as acceptable (clinically usable), unacceptable, or misclassified. The clinical usability of the segmentations was determined based on whether additional mask edits would be required and whether the resulting flow curves were physiologically consistent across vessels within a patient, as assessed by the expert, thus reflecting standard clinical practice.

### Deep temporal clustering model

#### Model architecture

Unsupervised clustering of flow curves was performed using Deep Temporal Clustering ^24^. The model consisted of a temporal autoencoder and a temporal clustering layer (Fig. 1).

##### Temporal autoencoder

The autoencoder reduced the dimensionality of the input flow curve data to a latent space representation (*z*_*i*_) that preserved key temporal features^48^. The encoder consisted of a 1D convolutional layer (50 filters, kernel size = 10), a 1D max pool (pool size = 3), and two bidirectional long short-term memory layers (50 and 1 units) . The decoder reconstructed sequences using a time-distributed fully connected layer (50 filters), followed by upsampling and a deconvolutional layer (kernel size = 10).

##### Temporal clustering layer

This layer clusters the latent vectors, *z*_*i*_, from the autoencoder, into groups with similar temporal patterns^49^. It first initialized *k* centroids via *k*-means on *z*_*i*_, then computed soft cluster probability assignments (*q*_*ij*_) based on the Euclidean distance between each *z*_*i*_ and cluster centroid *μ*_*j*_, using Student’s t-distribution, defined as ^25^:

$${\mathrm{q}}_{{{\mathrm{ij}}}} = \frac{{\left( {1 + \left| {\left| {{\mathrm{z}}_{{\mathrm{i}}} - \mu_{{\mathrm{j}}} } \right|} \right|^{2} } \right)^{ - 1} }}{{\Sigma_{{{\mathrm{j}}^{\prime}}} \left( {1 + \left| {\left| {{\mathrm{z}}_{{\mathrm{i}}} - \mu_{{{\mathrm{j}}^{\prime}}} } \right|} \right|^{2} } \right)^{ - 1} }}$$

The clustering layer then self-trains by iteratively minimizing the Kullback–Leibler (KL) divergence between the soft assignments (*q*_*ij*_) and a target distribution (*p*_*ij*_), resulting in higher-confidence and more distinct clusters. The target distribution was defined as ^49^:

$${\mathrm{p}}_{{{\mathrm{ij}}}} = \frac{{\left( {{\mathrm{q}}_{{{\mathrm{ij}}}}^{2} {/}\Sigma_{{\mathrm{i}}} {\mathrm{q}}_{{{\mathrm{ij}}}} } \right)}}{{\Sigma_{{{\mathrm{j}}^{\prime}}} \left( {{\mathrm{q}}_{{{\mathrm{ij}}^{\prime}}}^{2} {/}\Sigma_{{\mathrm{i}}} {\mathrm{q}}_{{{\mathrm{ij}}^{\prime}}} } \right)}}$$

#### Training data

The DTC model was trained using flow curve data derived from DCS-based segmentations. Only data from CMR exams with acceptable aortic segmentations were included. Exams were also excluded if: (i) The aortic flow curve peaked in the second half of the cardiac cycle (N = 21) as they were likely pulse-gated, since pulse-gated data cannot be aligned in the diastolic frame with the rest of the data which is ECG-gated, (ii) The aortic flow was non-physiological (< 10 mL; N = 7), or (iii) The nominal interval (N = 16) or BSA (N = 196) data were missing.

After exclusions, 2416 exams had acceptable aortic segmentations and complete demographic data; of these, 1943 had acceptable LPA/RPA and 2286 had acceptable SVC/IVC segmentations.

To have consistent data for clustering, all flow curves were BSA-indexed and cubic-spline interpolated to 30 frames (median frames across all PCMR series in the pipeline). Each flow curve was z-score normalized by subtracting the mean and dividing by the standard deviation of the whole dataset. Processed flow curves from the LPA/RPA or SVC/IVC were then concatenated and treated as separate channels.

#### Model training

Our DTC model was trained following the original methodology described by Madiraju et al. ^24^. To establish meaningful latent representations before clustering, the autoencoder was pretrained for 100 epochs with a learning rate of 0.001, minimizing the mean square error between the input and the reconstruction. The initial latent space of the autoencoder was used to initialize cluster centroids in the temporal clustering layer to create soft cluster labels. The autoencoder and clustering layer were then optimized jointly using the Adam optimizer (learning rate = 0.001), minimizing both mean squared error for accurate latent space representation and the KL divergence to increase cluster clarity^50^, with both losses equally weighted. Cluster assignments were updated every 100 epochs until convergence was reached, defined as fewer than 0.1% of samples changing cluster assignments.

We trained two DTC models for the branch pulmonary arteries (DTC_PA_) and for the vena cavae (DTC_VC_). Both models shared the same architecture and training parameters. As DTC is fully unsupervised, the entire dataset was used for both training and inference.

To determine the optimal number of clusters, we performed a sensitivity analysis varying from 3–8 clusters. The optimal number of clusters was based on maximizing the temporal silhouette score (which quantifies intra-cluster cohesion and inter-cluster separation^51^) and demonstrating significant differences for death/transplantation and liver outcomes using time-varying cox regression.

To assess temporal changes in the patients’ flow profiles, we also measured the changes in the cluster assignments identified by the DTC models across multiple scans over time for patients with multiple scans.

#### Comparison to PCA-based clustering

As DTC is a more complex deep learning methodology, we also compared our method with conventional temporal PCA followed by *k*-means clustering to see if the observed flow patterns in the centroid clusters generated were consistent across methods.

To ensure consistency, the training data used to train the DTC models was kept the same for the PCA models (PA model: N = 1943 and VC model: N = 2286). As with the DTC model, all flow curves were interpolated to a consistent 30 timepoints. For each model, the vessel flow curves were concatenated end-to-end to create a 60-dimensional feature vector (LPA/RPA for the PA model and SVC/IVC for the VC model). The data was then centered at the mean by subtracting the mean of the whole dataset from each flow curve, separately for the PA and VC models. PCA was performed to obtain PC weights for each data sample. These weights were then used as input into the *k*-means clustering model, using the same number of clusters as the DTC model.

### Statistical analysis

Continuous variables are expressed as medians with IQRs, as most variables were not normally distributed. Statistical analyses were performed using the SciPy (version 1.9.0), scikit-posthocs (version 0.11.2), pengouin (version 0.5.5), and lifelines (version 0.27.8) libraries in Python, and *p* < 0.05 was considered statistically significant.

To compare patient demographics across train, validation, and test ground truth datasets, as well as between patient clusters identified by DTC models, we used the Kruskal–Wallis test with post-hoc Dunn testing (Benjamini/Hochberg correction) for continuous variables. For categorical variables, we applied the χ2 test followed by post-hoc pairwise χ^2^ comparisons.

#### Segmentation performance analysis

For evaluation of the DCS model on the test set (N = 50 exams), Kruskal–Wallis and post-hoc Dunn (Benjamini/Hochberg correction) tests were applied to identify statistical differences in Dice score across vessels. Intraclass correlation and Bland–Altman analysis assessed agreement between net forward volumes from DL and manual segmentations. Wilcoxon signed-rank tests were used to assess the significance of differences between the DL and manually derived net forward volumes.

For evaluation on the FORCE registry (N = 2902 exams), we used χ^2^ tests to compare the categorical variables that influenced the segmentation success (Table 1).

#### Cluster outcome analysis

To evaluate the prognostic significance of flow phenotype clusters derived from our DTC models, we assessed their association with two clinical outcomes: (i) Death or transplantation and (ii) First diagnosis of Fontan-associated liver disease, the number of patients with these outcomes are shown in Supplementary Table 9. We applied the same methodology to the clusters found using PCA-based clustering for comparison.

Since many patients underwent multiple exams, clusters were assigned independently for each exam. Time-dependent Cox proportional hazards models were used to estimate risk, adjusting for age, sex, body surface area (BSA), indexed aortic flow rate, and ejection fraction to control for potential confounders. Each cluster was used once as the reference group when calculating hazard ratios.

Risk was visualized using Simon-Makuch plots, which extend Kaplan–Meier curves to incorporate time-varying covariates. Plots were truncated when fewer than ten individuals remained at risk in any cluster to preserve interpretability.

To explore potential explanations for differences in survival, we compared patient characteristics and clinical metrics across the identified flow phenotypes. Patient characteristics included age, sex, body surface area, type of Fontan procedure, type of systemic ventricular circulation, presence of heterotaxy, situs type, and diagnosis. Clinical metrics obtained from the same session as the PCMR included end-diastolic volume, end-systolic volume, and ejection fraction, all derived from short-axis cine imaging. PCMR-specific measures included collateral flow, the distribution of flow across vessels, and the flow rate in each of five key vessels. The data used in this study was from the Fontan Outcomes Registry using CMR Examinations (FORCE registry), http://www.forceregistry.org, which is available to members of the registry.

## Supplementary Information

Supplementary Information.

## Data Availability

The data used in this study is only available for access for institutes with an agreement with the FORCE registry. For inquiries, contact the FORCE registry’s principal investigator, Rahul H. Rathod, at rahul.rathod@childrens.harvard.edu.

## Code availability

The code for the implementation of the DCS and DTC models has been made publicly available: https://github.com/Ti-Yao/MultiFlow.
