# Supplementary material for: Deep learning for vessel segmentation and flow analysis to identify clusters associated with adverse outcomes in a fontan patient registry
Source: Sci Rep. 2026 Mar 4;16:11956. doi: 10.1038/s41598-026-40738-6 (PMC13068955; doi:10.1038/s41598-026-40738-6)
Supplement: Supplementary file 1 — Supplementary Information. [file 41598_2026_40738_MOESM1_ESM.docx]

# Supplementary Information

Supplementary Table 1: Patient demographics for the ground truth data used to train, validate and test the Deep Classification + Segmentation (DCM) model. Continuous values are quoted as median (interquartile range) and categorical variables are number (percentage). Demographics for the patient exams used for DCS training (N = 175), validation (N=25), testing (N=50) and the combined dataset (N = 260). Columns may not sum to totals due to some missing demographic data.

|  | **No. of Patients Exams in Training Dataset (N = 185)** | **No. of Patients Exams in Validation Dataset (N = 25)** | **No. of Patients Exams in Testing Dataset (N = 50)** | **No. of Patients Exams in Manually Segmented Dataset (N = 260)** | **p** |
| --- | --- | --- | --- | --- | --- |
| **Age** | 17.1 (13.5–24.3) (N = 163) | 13.9 (12.8–15.8) (N = 21) | 16.7 (12.4–23.4) (N = 44) | 16.7 (13.1–23.8) (N = 228) | 0.108 |
| **Adult** | 76 (41) | 5 (20) | 19 (38) | 100 (38) | 0.139 |
| **Child (<18)** | 87 (47) | 16 (64) | 25 (50) | 128 (49) |  |
| **BSA (m^2^)** | 1.6 (1.3–1.8) (N = 163) | 1.5 (1.3–1.7) (N = 21) | 1.7 (1.2–1.9) (N = 44) | 1.6 (1.3–1.9) (N = 228) | 0.644 |
| **Heart Rate (BPM)** | 77.0 (67.0–87.0) (N = 142) | 72.0 (57.0–84.0) (N = 18) | 78.0 (66.0–90.0) (N = 35) | 77.0 (66.0–87.0) (N = 195) | 0.429 |
| **EDV_i_ (mL/m^2^)** | 102.1 (84.3–126.2) (N = 163) | 108.9 (79.7–130.2) (N = 21) | 104.0 (80.6–126.9) (N = 44) | 103.2 (81.8–126.4) (N = 228) | 0.99 |
| **ESV_i_ (mL/m^2^)** | 50.5 (37.8–69.2) (N = 163) | 53.9 (38.3–65.9) (N = 21) | 54.4 (36.5–66.8) (N = 44) | 51.3 (37.1–67.8) (N = 228) | 0.951 |
| **EF (%)** | 51.0 (43.0–56.0) (N = 163) | 52.0 (48.0–57.0) (N = 21) | 52.0 (45.0–56.0) (N = 44) | 51.0 (44.0–56.0) (N = 228) | 0.686 |
| **Sex** |  |  |  |  |  |
| **Male** | 97 (52) | 11 (44) | 26 (52) | 134 (52) | 0.822 |
| **Female** | 66 (36) | 10 (40) | 18 (36) | 94 (36) |  |
| **Bilateral Aorta** | 12 (6) | 0 (0) | 5 (10) | 17 (7) | 0.255 |
| **Bilateral SVC** | 15 (8) | 3 (12) | 6 (12) | 24 (9) | 0.617 |
| **Fontan Type** |  |  |  |  | 0.064 |
| **Lateral** | 78 (42) | 4 (16) | 15 (30) | 97 (37) |  |
| **Extracardiac** | 69 (37) | 14 (56) | 25 (50) | 108 (42) |  |
| **Other Type** | 12 (6) | 3 (12) | 4 (8) | 19 (7) |  |
| **Systemic Ventricular Circulation** |  |  |  |  | 0.872 |
| **Both** | 94 (51) | 13 (52) | 24 (48) | 131 (50) |  |
| **Right** | 42 (23) | 6 (24) | 11 (22) | 59 (23) |  |
| **Left** | 27 (15) | 2 (8) | 9 (18) | 38 (15) |  |
| **Diagnosis** |  |  |  |  | 0.877 |
| **HLHS** | 52 (28) | 8 (32) | 13 (26) | 73 (28) |  |
| **Tricuspid Atresia** | 25 (14) | 3 (12) | 8 (16) | 36 (14) |  |
| **Double Outlet Right Ventricle** | 16 (9) | 1 (4) | 6 (12) | 23 (9) |  |
| **Double Inlet Left Ventricle** | 26 (14) | 2 (8) | 4 (8) | 32 (12) |  |
| **Other Diagnosis** | 44 (24) | 7 (28) | 13 (26) | 64 (25) |  |
| **Heterotaxy** |  |  |  |  | 0.903 |
| **No** | 140 (76) | 18 (72) | 36 (72) | 194 (75) |  |
| **Yes** | 22 (12) | 3 (12) | 7 (14) | 32 (12) |  |
| **Situs Type** |  |  |  |  | 0.178 |
| **Levocardiac** | 169 (91) | 24 (96) | 42 (84) | 235 (90) |  |
| **Dextrocardiac** | 16 (9) | 1 (4) | 8 (16) | 25 (10) |  |
| **Mesocardiac** | 0 (0) | 0 (0) | 0 (0) | 0 (0) |  |

Supplementary Table 2: Patient demographics for ground truth dataset, pipeline analysis and the whole FORCE registry. Continuous values are quoted as median (interquartile range) and categorical variables are number (percentage).Demographics for the patient exams used for DCS training/validation (N = 260), for evaluation of the pipeline (N = 2902) and the exams in the whole registry that have phase-contrast series (N = 4881). Situs type was manually identified in the training/validation and pipeline datasets but not the whole registry. Columns may not sum to totals due to some missing demographic data.

|  | **No. of Patients Exams in Manually Segmented Dataset (N = 260)** | **No. of Patients Exams in Pipeline Validated Dataset (N = 2902)** | **No. of Exams with Phase-Contrast in FORCE registry (N = 4881)** |
| --- | --- | --- | --- |
| **Age** | 16.7 (13.1–23.8) (N = 228) | 15.1 (10.6–20.6) (N = 2712) | 15.8 (11.3–21.9) (N = 4485) |
| **Adult** | 100 (38) | 909 (31) | 1727 (35) |
| **Child (<18)** | 128 (49) | 1803 (62) | 2758 (57) |
| **BSA (m^2^)** | 1.6 (1.3–1.9) (N = 228) | 1.5 (1.1–1.8) (N = 2704) | 1.5 (1.2–1.8) (N = 4469) |
| **Heart Rate (BPM)** | 77.0 (66.0–87.0) (N = 195) | 79.0 (68.0–91.0) (N = 2045) | 79.0 (68.0–91.0) (N = 3465) |
| **EDV_i_ (mL/m^2^)** | 103.2 (81.8–126.4) (N = 228) | 98.9 (80.3–123.7) (N = 2665) | 99.1 (80.0–124.5) (N = 4387) |
| **ESV_i_ (mL/m^2^)** | 51.3 (37.1–67.8) (N = 228) | 47.0 (35.2–63.2) (N = 2659) | 47.2 (35.1–64.4) (N = 4364) |
| **EF (%)** | 51.0 (44.0–56.0) (N = 228) | 52.0 (46.0–58.0) (N = 2663) | 52.0 (45.0–58.0) (N = 4373) |
| **Sex** |  |  |  |
| **Male** | 134 (52) | 1637 (56) | 2684 (55) |
| **Female** | 94 (36) | 1075 (37) | 1801 (37) |
| **Bilateral Aorta** | 17 (7) | 339 (12) | – |
| **Bilateral SVC** | 24 (9) | 377 (13) | – |
| **Fontan Type** |  |  |  |
| **Lateral** | 97 (37) | 1216 (42) | 2021 (41) |
| **Extracardiac** | 108 (42) | 1206 (42) | 1905 (39) |
| **Other Type** | 19 (7) | 252 (9) | 501 (10) |
| **Systemic Ventricular Circulation** |  |  |  |
| **Both** | 131 (50) | 1399 (48) | 2359 (48) |
| **Right** | 59 (23) | 810 (28) | 1249 (26) |
| **Left** | 38 (15) | 499 (17) | 868 (18) |
| **Diagnosis** |  |  |  |
| **HLHS** | 73 (28) | 950 (33) | 1443 (30) |
| **Tricuspid Atresia** | 36 (14) | 451 (16) | 746 (15) |
| **Double Outlet Right Ventricle** | 23 (9) | 310 (11) | 542 (11) |
| **Double Inlet Left Ventricle** | 32 (12) | 296 (10) | 529 (11) |
| **Other Diagnosis** | 64 (25) | 703 (24) | 1223 (25) |
| **Heterotaxy** |  |  |  |
| **Yes** | 32 (12) | 294 (10) | 560 (11) |
| **No** | 194 (75) | 2410 (83) | 3907 (80) |
| **Situs Type** |  |  |  |
| **Levocardiac** | 235 (90) | 2675 (92) | – |
| **Dextrocardiac** | 25 (10) | 202 (7) | – |
| **Mesocardiac** | 0 (0) | 25 (1) | – |

Supplementary Table 3: Cluster Sensitivity Analysis. Deep Temporal Clustering (DTC) models for the pulmonary arteries (PA) and vena cavae (VC) were evaluated across multiple values of k. For each k, silhouette score and statistical differences between identified clusters for clinical outcomes were assessed. Rows shown in orange indicate the chosen k values. Rows in gray indicate values of k for which the DTC model failed to identify the specified number of clusters.

| **DTC Model** | **Number of clusters, *k*** | **Silhouette**  **Score** | **Statistical Differences in Adverse Outcomes between Clusters** | |
| --- | --- | --- | --- | --- |
|  |  |  | **Death / Transplantation** | **Liver Disease** |
| PA | 3 | 0.84 | No | No |
|  | 4 | 0.84 | No | No |
|  | **5** | **0.89** | **Yes** | No |
|  | 6 | 0.82 | No | **Yes** |
|  | 7 | 0.75 | No | No |
|  | 8 | - | - | - |
| VC | 3 | 0.89 | **Yes** | No |
|  | **4** | **0.90** | **Yes** | **Yes** |
|  | 5 | - | - | - |

Supplementary Table 4: Full demographics for the patients in the 5 identified clusters identified by DTC_PA_. Continuous values are quoted as median (interquartile range) and categorical variables are number (percentage). HLHS – Hypoplastic Left Heart Syndrome, EDV_i_ – indexed end-diastolic volume, ESV_i_  – indexed end-systolic volume, EF – ejection fraction, LPA – Left Pulmonary Artery, RPA – Right Pulmonary Artery, AO – Aorta, SVC – Superior Vena Cava, IVC – Inferior Vena cava

|  | **All** **(N = 1943)** | **1: Normal Distribution \| High Flow** **(N = 610)** | **2: Normal Distribution \| Low Flow** **(N = 193)** | **3: Diastolic-Dominant \| Normal Flow** **(N = 253)** | **4: RPA-Dominant \| Normal Flow** **(N = 267)** | **5: Balanced Distribution \| Normal Flow** **(N = 620)** | **p** |
| --- | --- | --- | --- | --- | --- | --- | --- |
| **Total PA Flow** | 2.33 (1.92–2.73) | 2.91 (2.59–3.28) | 1.42 (1.21–1.67) | 1.96 (1.72–2.12) | 2.34 (2.16–2.59) | 2.18 (1.88–2.45) |  |
| **Ratio of LPA Flow to Total PA Flow** | 0.43 (0.37–0.5) | 0.42 (0.37–0.45) | 0.45 (0.41–0.49) | 0.38 (0.33–0.41) | 0.3 (0.23–0.35) | 0.52 (0.48–0.58) | **<0.001** |
| **Scan Year** | 2018  (2015–2021) | 2018  (2015–2021) | 2018  (2015–2021) | 2018  (2015–2021) | 2017  (2015–2020) | 2018  (2015–2021) | 0.18 |
| **Age** | 15.3 (11.1–20.8) | 13.7 (10.4–18.4) | 17.0 (11.4–23.3) | 16.6 (12.6–22.5) | 14.5 (10.8–19.3) | 16.1 (12.0–22.8) | **<0.001** |
| **Adult** | 679 (35) | 160 (26) | 88 (46) | 102 (40) | 81 (30) | 248 (40) | **<0.001** |
| **Child (<18)** | 1264 (65) | 450 (74) | 105 (54) | 151 (60) | 186 (70) | 372 (60) |  |
| **BSA (m^2^)** | 1.5 (1.2–1.8) | 1.4 (1.1–1.7) | 1.6 (1.2–1.8) | 1.6 (1.3–1.8) | 1.5 (1.0–1.8) | 1.5 (1.2–1.8) | **<0.001** |
| **Heart Rate (BPM)** | 77 (66–89) | 82 (71–92) | 74 (66–88) | 70 (63–85) | 78 (68–91) | 74 (64–84) | **<0.001** |
| **EDV_i_**  **(mL/m^2^)** | 99.2 (80.3–123.7) (N = 1915) | 101.5 (84.2–124.0) (N = 595) | 105.6 (82.3–135.5) (N = 192) | 102.8 (82.3–125.9) | 102.5 (84.8–130.8) (N = 265) | 94.5 (76.1–115.4) (N = 610) | **<0.001** |
| **ESV_i_ (mL/m^2^)** | 47.5 (35.7–63.7) (N = 1911) | 47.5 (36.5–62.2) (N = 594) | 54.0 (39.5–73.0) (N = 191) | 51.3 (37.5–66.8) (N = 251) | 49.8 (35.9–67.2) (N = 265) | 44.4 (32.5–58.7) (N = 610) | **<0.001** |
| **EF (%)** | 52 (45–57) (N = 1911) | 53 (47–58) (N = 594) | 49 (42–53) (N = 191) | 51 (45–56) (N = 251) | 52 (44–59) (N = 265) | 53 (45–58) (N = 610) | **<0.001** |
| **Indexed AO Flow Rate (L/min/m^2^)** | 3.1 (2.6–3.6) | 3.5 (3.0–4.0) | 2.5 (2.1–3.1) | 2.8 (2.4–3.3) | 3.2 (2.7–3.7) | 2.9 (2.5–3.3) | **<0.001** |
| **Indexed LPA Flow Rate (L/min/m^2^)** | 1.0 (0.8–1.2) | 1.2 (1.0–1.4) | 0.7 (0.5–0.8) | 0.7 (0.6–0.9) | 0.7 (0.5–0.8) | 1.1 (1.0–1.3) | **<0.001** |
| **Indexed RPA Flow Rate (L/min/m^2^)** | 1.3 (1.0–1.6) | 1.7 (1.5–1.9) | 0.8 (0.7–0.9) | 1.2 (1.1–1.3) | 1.7 (1.5–1.9) | 1.0 (0.9–1.2) | **<0.001** |
| **Indexed SVC Flow Rate (L/min/m^2^)** | 0.9 (0.7–1.2) (N = 1887) | 1.1 (0.9–1.3) (N = 592) | 0.7 (0.6–0.9) (N = 186) | 0.8 (0.6–1.0) (N = 246) | 0.9 (0.7–1.2) (N = 259) | 0.8 (0.7–1.1) (N = 604) | **<0.001** |
| **Indexed IVC Flow Rate (L/min/m^2^)** | 1.6 (1.2–1.9) (N = 1907) | 1.9 (1.5–2.2) (N = 604) | 1.1 (0.9–1.4) (N = 186) | 1.4 (1.1–1.7) (N = 249) | 1.5 (1.1–1.8) (N = 263) | 1.5 (1.2–1.8) (N = 605) | **<0.001** |
| **Collateral (%)** | 21 (12–32) (N = 1523) | 18 (10–28) (N = 463) | 25 (17–39) (N = 161) | 23 (14–34) (N = 211) | 23 (13–36) (N = 216) | 19 (11–32) (N = 472) | **<0.001** |
| **Sex** |  |  |  |  |  |  |  |
| **Male** | 1175 (60) | 395 (65) | 102 (53) | 152 (60) | 174 (65) | 352 (57) | **0.004** |
| **Female** | 768 (40) | 215 (35) | 91 (47) | 101 (40) | 93 (35) | 268 (43) |  |
| **Fontan Type** |  |  |  |  |  |  | **<0.001** |
| **Lateral** | 865 (45) | 280 (46) | 79 (41) | 128 (51) | 141 (53) | 237 (38) |  |
| **Extracardiac** | 871 (45) | 283 (46) | 92 (48) | 109 (43) | 115 (43) | 272 (44) |  |
| **Other Type** | 207 (11) | 47 (8) | 22 (11) | 16 (6) | 11 (4) | 111 (18) |  |
| **Systemic Ventricular Circulation** |  |  |  |  |  |  | 0.05 |
| **Both** | 956 (49) | 300 (49) | 106 (55) | 123 (49) | 127 (48) | 300 (48) |  |
| **Right** | 612 (31) | 199 (33) | 61 (32) | 86 (34) | 92 (34) | 174 (28) |  |
| **Left** | 372 (19) | 110 (18) | 26 (13) | 44 (17) | 47 (18) | 145 (23) |  |
| **Diagnosis** |  |  |  |  |  |  | **<0.001** |
| **HLHS** | 703 (36) | 234 (38) | 74 (38) | 99 (39) | 117 (44) | 179 (29) |  |
| **Tricuspid Atresia** | 346 (18) | 94 (15) | 29 (15) | 43 (17) | 39 (15) | 141 (23) |  |
| **Double Outlet Right Ventricle** | 208 (11) | 65 (11) | 26 (13) | 37 (15) | 22 (8) | 58 (9) |  |
| **Double Inlet Left Ventricle** | 234 (12) | 94 (15) | 11 (6) | 25 (10) | 33 (12) | 71 (11) |  |
| **Other Diagnosis** | 452 (23) | 123 (20) | 53 (27) | 49 (19) | 56 (21) | 171 (28) |  |
| **Heterotaxy** |  |  |  |  |  |  | **<0.001** |
| **No** | 1783 (92) | 571 (94) | 169 (88) | 236 (93) | 258 (97) | 549 (89) |  |
| **Yes** | 151 (8) | 36 (6) | 23 (12) | 17 (7) | 8 (3) | 67 (11) |  |
| **Situs Type** |  |  |  |  |  |  | **0.002** |
| **Levocardiac** | 1835 (94) | 581 (95) | 182 (94) | 245 (97) | 261 (98) | 566 (91) |  |
| **Dextrocardiac** | 90 (5) | 22 (4) | 9 (5) | 6 (2) | 5 (2) | 48 (8) |  |
| **Mesocardiac** | 18 (1) | 7 (1) | 2 (1) | 2 (1) | 1 (0) | 6 (1) |  |
| **VC Clusters** |  |  |  |  |  |  |  |
| **Cluster 1** | 582 (30) | 110 (18) | 68 (35) | 111 (44) | 86 (32) | 207 (33) |  |
| **Cluster 2** | 415 (21) | 176 (29) | 28 (15) | 35 (14) | 69 (26) | 107 (17) |  |
| **Cluster 3** | 469 (24) | 219 (36) | 19 (10) | 31 (12) | 63 (24) | 137 (22) |  |
| **Cluster 4** | 385 (20) | 81 (13) | 64 (33) | 65 (26) | 37 (14) | 138 (22) |  |
| **N/A** | 92 (5) | 24 (4) | 14 (7) | 11 (4) | 12 (4) | 31 (5) |  |

Supplementary Table 5. Clinical and demographic differences (p < 0.05) across five clusters predicted by DTC_PA_. HLHS – Hypoplastic Left Heart Syndrome, EDVi – indexed end-diastolic volume, ESVi – indexed end-systolic volume, EF – ejection fraction, LPA – Left Pulmonary Artery, RPA – Right Pulmonary Artery, AO – Aorta, SVC – Superior Vena Cava, IVC – Inferior Vena Cava. Clusters: (1) Normal Distribution | High Flow, (2) Balanced Distribution | Low Flow, (3) Diastolic-Dominant | Normal Flow, (4) RPA-Dominant | Normal Flow, (5) Balanced Distribution | Normal Flow.

| **Variable** | **Overall** | **1 vs 2** | **1 vs 3** | **1 vs 4** | **1 vs 5** | **2 vs 3** | **4 vs 2** | **4 vs 3** | **4 vs 5** | **5 vs 2** | **5 vs 3** |
| --- | --- | --- | --- | --- | --- | --- | --- | --- | --- | --- | --- |
| **Ratio of LPA Flow to Total PA Flow** | **<0.001** | **<0.001** | **<0.001** | **<0.001** | **<0.001** | **<0.001** | **<0.001** | **<0.001** | **<0.001** | **<0.001** | **<0.001** |
| **Scan Year** | 0.179 | 0.292 | 0.739 | 0.396 | 0.637 | 0.292 | 0.182 | 0.556 | 0.292 | 0.396 | 0.556 |
| **Age** | **<0.001** | **<0.001** | **<0.001** | 0.8 | **<0.001** | 0.968 | **0.002** | **0.001** | **<0.001** | 0.968 | 0.968 |
| **Adult** | **<0.001** | **<0.001** | **<0.001** | 0.241 | **<0.001** | 0.307 | **0.001** | **0.022** | **0.008** | 0.195 | 0.992 |
| **BSA (m2)** | **<0.001** | **0.017** | **0.001** | 0.696 | **<0.001** | 0.696 | 0.108 | **0.017** | **0.017** | 0.79 | 0.718 |
| **Heart Rate (BPM)** | **<0.001** | **<0.001** | **<0.001** | 0.05 | **<0.001** | 0.087 | 0.05 | **<0.001** | **<0.001** | 0.246 | 0.33 |
| **EDV_i_**  **(mL/m^2^)** | **<0.001** | 0.429 | 0.934 | 0.548 | **<0.001** | 0.429 | 0.746 | 0.548 | **<0.001** | **<0.001** | **0.002** |
| **ESV_i_**  **(mL/m^2^)** | **<0.001** | **0.002** | 0.16 | 0.342 | **0.005** | 0.122 | 0.052 | 0.656 | **0.002** | **<0.001** | **0.001** |
| **EF (%)** | **<0.001** | **<0.001** | **<0.001** | 0.168 | 0.416 | **0.03** | **<0.001** | **0.04** | 0.416 | **<0.001** | **0.001** |
| **Indexed AO Flow Rate**  **(L/min/m^2^)** | **<0.001** | **<0.001** | **<0.001** | **<0.001** | **<0.001** | **0.001** | **<0.001** | **<0.001** | **<0.001** | **<0.001** | 0.099 |
| **Indexed LPA Flow Rate**  **(L/min/m^2^)** | **<0.001** | **<0.001** | **<0.001** | **<0.001** | **0.001** | **0.022** | 0.178 | 0.282 | **<0.001** | **<0.001** | **<0.001** |
| **Indexed RPA Flow Rate**  **(L/min/m^2^)** | **<0.001** | **<0.001** | **<0.001** | 0.34 | **<0.001** | **<0.001** | **<0.001** | **<0.001** | **<0.001** | **<0.001** | **<0.001** |
| **Indexed SVC Flow Rate**  **(L/min/m^2^)** | **<0.001** | **<0.001** | **<0.001** | **<0.001** | **<0.001** | **0.022** | **<0.001** | **<0.001** | **<0.001** | **<0.001** | **0.003** |
| **Indexed IVC Flow Rate**  **(L/min/m^2^)** | **<0.001** | **<0.001** | **<0.001** | **<0.001** | **<0.001** | **<0.001** | **<0.001** | 0.051 | 0.346 | **<0.001** | **0.001** |
| **Collateral (%)** | **<0.001** | **<0.001** | **0.001** | **<0.001** | **0.037** | 0.06 | 0.136 | 0.59 | **0.024** | **<0.001** | 0.079 |
| **Male** | **0.004** | **0.004** | 0.222 | 0.967 | **0.005** | 0.152 | **0.01** | 0.268 | **0.024** | 0.381 | 0.411 |
| **Fontan Type** | **<0.001** | 0.087 | 0.538 | 0.178 | **<0.001** | 0.086 | **0.005** | 0.494 | **<0.001** | 0.103 | **<0.001** |
| **Systemic Ventricular Circulation** | 0.05 | 0.246 | 0.926 | 0.858 | **0.041** | 0.348 | 0.263 | 0.98 | 0.065 | **0.012** | 0.078 |
| **Diagnosis** | **<0.001** | **0.007** | 0.154 | 0.435 | **<0.001** | 0.31 | **0.035** | 0.157 | **<0.001** | **0.003** | **0.001** |
| **Heterotaxy** | **<0.001** | **0.008** | 0.777 | 0.099 | **0.003** | 0.079 | **<0.001** | 0.077 | **<0.001** | 0.77 | 0.079 |
| **Situs Type** | **0.002** | 0.797 | 0.575 | 0.207 | **0.007** | 0.395 | 0.153 | 0.758 | **0.002** | 0.343 | **0.011** |

Supplementary Table 6: Full demographics for the patients in the 4 identified clusters identified by DTC_VC_. Continuous values are quoted as median (interquartile range) and categorical variables are number (percentage). HLHS – Hypoplastic Left Heart Syndrome, EDV_i_ – indexed end-diastolic volume, ESV_i_  – indexed end-systolic volume, EF – ejection fraction, LPA – Left Pulmonary Artery, RPA – Right Pulmonary Artery, AO – Aorta, SVC – Superior Vena Cava, IVC – Inferior Vena Cava.

|  | **All (N = 2286)** | **1: IVC-Dominant \| Normal Flow (N = 711)** | **2: SVC-Dominant \| High Flow (N = 553)** | **3: Normal Distribution \| High Flow (N = 558)** | **4: Normal Distribution \| Normal Flow (N = 464)** | **p** |
| --- | --- | --- | --- | --- | --- | --- |
| **Total VC Flow** | 2.50 (2.10–2.98) | 2.32 (2.02–2.63) | 2.83 (2.42–3.33) | 2.86 (2.44–3.25) | 2.15 (1.75–2.52) |  |
| **Ratio of SVC Flow to Total VC Flow** | 0.36 (0.30–0.47) | 0.28 (0.24–0.31) | 0.56 (0.48–0.65) | 0.37 (0.33–0.43) | 0.37 (0.32–0.41) | **<0.001** |
| **Scan Year** | 2018 (2015–2021) | 2019 (2015–2021) | 2016 (2014–2019) | 2018 (2016–2021) | 2018 (2015–2021) | **<0.001** |
| **Age** | 15.3 (10.8–20.6) | 20.6 (16.7–26.5) | 8.2 (5.4–10.8) | 13.5 (11.3–16.4) | 17.2 (14.3–22.5) | **<0.001** |
| **Adult** | 778 (34) | 467 (66) | 14 (3) | 92 (16) | 205 (44) | **<0.001** |
| **Child (<18)** | 1508 (66) | 244 (34) | 539 (97) | 466 (84) | 259 (56) |  |
| **BSA (m^2^)** | 1.5 (1.1–1.8) | 1.8 (1.6–2.0) | 0.9 (0.7–1.1) | 1.4 (1.2–1.6) | 1.6 (1.4–1.8) | **<0.001** |
| **Heart Rate (BPM)** | 77 (67–89) | 71 (63–80) | 90 (79–105) | 80 (71–90) | 71 (62–81) | **<0.001** |
| **EDVi (mL/m^2^)** | 99.5 (80.8–123.9) (N = 2262) | 98.8 (79.7–123.2) (N = 708) | 98.6 (79.9–123.6) (N = 548) | 101.4 (81.9–122.0) (N = 544) | 99.7 (83.4–127.6) (N = 462) | 0.671 |
| **ESVi (mL/m^2^)** | 47.7 (35.8–64.0) (N = 2257) | 47.5 (34.8–64.9) (N = 708) | 46.7 (35.7–62.1) (N = 545) | 47.3 (36.3–62.2) (N = 542) | 50.2 (36.7–68.0) (N = 462) | 0.138 |
| **EF (%)** | 52 (45–57) (N = 2257) | 51 (45–57) (N = 708) | 53 (46–58) (N = 545) | 52 (45–58) (N = 542) | 51 (43–56) (N = 462) | **0.002** |
| **Indexed AO Flow Rate (L/min/m^2^)** | 3.1 (2.6–3.7) | 2.8 (2.4–3.2) | 3.7 (3.1–4.3) | 3.3 (2.9–3.7) | 2.7 (2.3–3.2) | **<0.001** |
| **Indexed LPA Flow Rate (L/min/m^2^)** | 1.0 (0.8–1.2) (N = 2118) | 0.9 (0.7–1.1) (N = 667) | 1.0 (0.8–1.3) (N = 492) | 1.1 (0.9–1.3) (N = 521) | 0.9 (0.7–1.1) (N = 438) | **<0.001** |
| **Indexed RPA Flow Rate (L/min/m^2^)** | 1.3 (1.0–1.7) (N = 1934) | 1.2 (0.9–1.5) (N = 599) | 1.4 (1.1–1.8) (N = 445) | 1.5 (1.2–1.8) (N = 493) | 1.2 (0.9–1.4) (N = 397) | **<0.001** |
| **Indexed SVC Flow Rate (L/min/m^2^)** | 0.9 (0.7–1.2) | 0.7 (0.5–0.7) | 1.5 (1.3–1.9) | 1.1 (1.0–1.1) | 0.8 (0.7–0.9) | **<0.001** |
| **Indexed IVC Flow Rate (L/min/m^2^)** | 1.5 (1.2–1.9) | 1.7 (1.4–1.9) | 1.2 (0.9–1.6) | 1.8 (1.4–2.1) | 1.4 (1.0–1.7) | **<0.001** |
| **Collateral (%)** | 21 (11–33) (N = 1887) | 20 (12–30) (N = 593) | 24 (14–36) (N = 468) | 18 (9–29) (N = 430) | 24 (13–38) (N = 396) | **<0.001** |
| **Sex** |  |  |  |  |  |  |
| **Male** | 1384 (61) | 417 (59) | 347 (63) | 355 (64) | 265 (57) | 0.082 |
| **Female** | 902 (39) | 294 (41) | 206 (37) | 203 (36) | 199 (43) |  |
| **Fontan Type** |  |  |  |  |  | **<0.001** |
| **Lateral** | 995 (44) | 401 (56) | 147 (27) | 242 (43) | 205 (44) |  |
| **Extracardiac** | 1056 (46) | 226 (32) | 358 (65) | 274 (49) | 198 (43) |  |
| **Other Type** | 235 (10) | 84 (12) | 48 (9) | 42 (8) | 61 (13) |  |
| **Systemic Ventricular Circulation** |  |  |  |  |  | **0.039** |
| **Both** | 1131 (49) | 379 (53) | 257 (46) | 273 (49) | 222 (48) |  |
| **Right** | 729 (32) | 197 (28) | 202 (37) | 172 (31) | 158 (34) |  |
| **Left** | 424 (19) | 135 (19) | 94 (17) | 111 (20) | 84 (18) |  |
| **Diagnosis** |  |  |  |  |  | 0.084 |
| **HLHS** | 830 (36) | 234 (33) | 232 (42) | 206 (37) | 158 (34) |  |
| **Tricuspid Atresia** | 380 (17) | 127 (18) | 85 (15) | 90 (16) | 78 (17) |  |
| **Double Outlet Right Ventricle** | 248 (11) | 72 (10) | 53 (10) | 68 (12) | 55 (12) |  |
| **Double Inlet Left Ventricle** | 255 (11) | 93 (13) | 55 (10) | 63 (11) | 44 (9) |  |
| **Other Diagnosis** | 573 (25) | 185 (26) | 128 (23) | 131 (23) | 129 (28) |  |
| **Heterotaxy** |  |  |  |  |  | **0.01** |
| **No** | 2033 (89) | 633 (89) | 485 (88) | 516 (92) | 399 (86) |  |
| **Yes** | 243 (11) | 74 (10) | 65 (12) | 41 (7) | 63 (14) |  |
| **Situs Type** |  |  |  |  |  | 0.56 |
| **Levocardiac** | 2084 (91) | 640 (90) | 508 (92) | 517 (93) | 419 (90) |  |
| **Dextrocardiac** | 180 (8) | 64 (9) | 41 (7) | 37 (7) | 38 (8) |  |
| **Mesocardiac** | 22 (1) | 7 (1) | 4 (1) | 4 (1) | 7 (2) |  |
| **PA Clusters** |  |  |  |  |  |  |
| **Cluster 1** | 586 (26) | 110 (15) | 176 (32) | 219 (39) | 81 (17) |  |
| **Cluster 2** | 179 (8) | 68 (10) | 28 (5) | 19 (3) | 64 (14) |  |
| **Cluster 3** | 242 (11) | 111 (16) | 35 (6) | 31 (6) | 65 (14) |  |
| **Cluster 4** | 255 (11) | 86 (12) | 69 (12) | 63 (11) | 37 (8) |  |
| **Cluster 5** | 589 (26) | 207 (29) | 107 (19) | 137 (25) | 138 (30) |  |
| **N/A** | 435 (19) | 129 (18) | 138 (25) | 89 (16) | 79 (17) |  |

Supplementary Table 7. Clinical and demographic differences (p < 0.05) across four clusters predicted by DTC_VC_. HLHS – Hypoplastic Left Heart Syndrome, EDVi – indexed end-diastolic volume, ESVi – indexed end-systolic volume, EF – ejection fraction, LPA – Left Pulmonary Artery, RPA – Right Pulmonary Artery, AO – Aorta, SVC – Superior Vena Cava, IVC – Inferior Vena Cava. Clusters: (1) IVC-Dominant | Normal Flow, (2) SVC-Dominant | High Flow, (3) Normal Distribution | High Flow, (4) Normal Distribution | Normal Flow.

| **Variable** | **Overall** | **1 vs 3** | **2 vs 1** | **2 vs 3** | **2 vs 4** | **4 vs 1** | **4 vs 3** |
| --- | --- | --- | --- | --- | --- | --- | --- |
| **Ratio of SVC Flow to Total VC Flow** | **<0.001** | **<0.001** | **<0.001** | **<0.001** | **<0.001** | **<0.001** | 0.168 |
| **Scan Year** | **<0.001** | 0.897 | **<0.001** | **<0.001** | **<0.001** | 0.897 | 0.897 |
| **Age** | **<0.001** | **<0.001** | **<0.001** | **<0.001** | **<0.001** | **<0.001** | **<0.001** |
| **Adult** | **<0.001** | **<0.001** | **<0.001** | **<0.001** | **<0.001** | **<0.001** | **<0.001** |
| **BSA (m^2^)** | **<0.001** | **<0.001** | **<0.001** | **<0.001** | **<0.001** | **<0.001** | **<0.001** |
| **Heart Rate (BPM)** | **<0.001** | **<0.001** | **<0.001** | **<0.001** | **<0.001** | 0.777 | **<0.001** |
| **EDV_i_ (mL/m^2^)** | 0.671 | 0.761 | 0.887 | 0.761 | 0.761 | 0.761 | 0.761 |
| **ESV_i_ (mL/m^2^)** | 0.138 | 0.989 | 0.635 | 0.635 | 0.145 | 0.199 | 0.199 |
| **EF (%)** | **0.002** | 0.45 | 0.119 | 0.418 | **0.001** | 0.05 | **0.016** |
| **Indexed AO Flow Rate (L/min/m^2^)** | **<0.001** | **<0.001** | **<0.001** | **<0.001** | **<0.001** | 0.283 | **<0.001** |
| **Indexed LPA Flow Rate (L/min/m^2^)** | **<0.001** | **<0.001** | **<0.001** | **0.001** | **<0.001** | 0.487 | **<0.001** |
| **Indexed RPA Flow Rate (L/min/m^2^)** | **<0.001** | **<0.001** | **<0.001** | 0.066 | **<0.001** | 0.265 | **<0.001** |
| **Indexed SVC Flow Rate (L/min/m^2^)** | **<0.001** | **<0.001** | **<0.001** | **<0.001** | **<0.001** | **<0.001** | **<0.001** |
| **Indexed IVC Flow Rate (L/min/m^2^)** | **<0.001** | **0.004** | **<0.001** | **<0.001** | **0.001** | **<0.001** | **<0.001** |
| **Collateral (%)** | **<0.001** | **0.008** | **<0.001** | **<0.001** | 0.719 | **0.002** | **<0.001** |
| **Male** | 0.082 | 0.081 | 0.155 | 0.811 | 0.078 | 0.644 | **0.04** |
| **Fontan Type** | **<0.001** | **<0.001** | **<0.001** | **<0.001** | **<0.001** | **<0.001** | **<0.001** |
| **Systemic Ventricular Circulation** | **0.039** | 0.31 | **0.004** | 0.117 | 0.7 | 0.064 | 0.522 |
| **Diagnosis** | 0.084 | 0.406 | **0.016** | 0.321 | 0.061 | 0.31 | 0.651 |
| **Heterotaxy** | **0.01** | 0.071 | 0.505 | **0.016** | 0.44 | 0.12 | **0.001** |
| **Situs Type** | 0.56 | 0.259 | 0.521 | 0.877 | 0.427 | 0.65 | 0.291 |

Supplementary Table 8: Data Dictionaries. Tunable Input Data Dictionary: One-hot encoding reference for tunable parameters. Reference Data Dictionary: Guide for selecting PC-MRI series when multiple acquisitions exist per vessel.

| **Vessel** | **Tunable Input Data Dictionary** | **Reference Data Dictionary** |
| --- | --- | --- |
| **LPA** | ['lpa', 'l pa', 'lt ', 'left'] | ['lpa'] |
| **RPA** | ['rpa', 'r pa', 'rt ', 'right'] | ['rpa'] |
| **Aorta** | ['aorta', 'ao', 'aor', 'asc', 'aso', 'neo', 'aa', 'aao', 'native', 'aov', 'av ', 'qs', 'stj', 'dks'] | ['asc','aa','aao','ao','neo','stj','dks'] |
| **SVC** | ['svc', 'rsvc', 'lsvc', 'sup', 'superior', 'glenn', 'vfc', 'bdg'] | ['svc'] |
| **IVC** | ['ivc', 'dao', 'inferior', 'fontan', 'font','inf '] | ['ivc'] |
| **Other** | ['', 'pv', 'avv', 'vein', 'lpv', 'rpv'] | - |

Supplementary Table 9. Clinical outcomes for the patient exams used in survival analysis. Patient counts for: (1) heart transplantation, (2) mortality, (3) composite death/transplant endpoint, and (4) liver disease comorbidity. The total number of patient exams analysed was N = 2902.

|  | Number of Patients in Dataset with Outcome | | | |
| --- | --- | --- | --- | --- |
|  | Transplant | Death | Transplant/Death | Liver Disease |
| DTC_PA_ | 22 | 41 | 62 | 204 |
| DTC_VC_ | 23 | 44 | 64 | 239 |


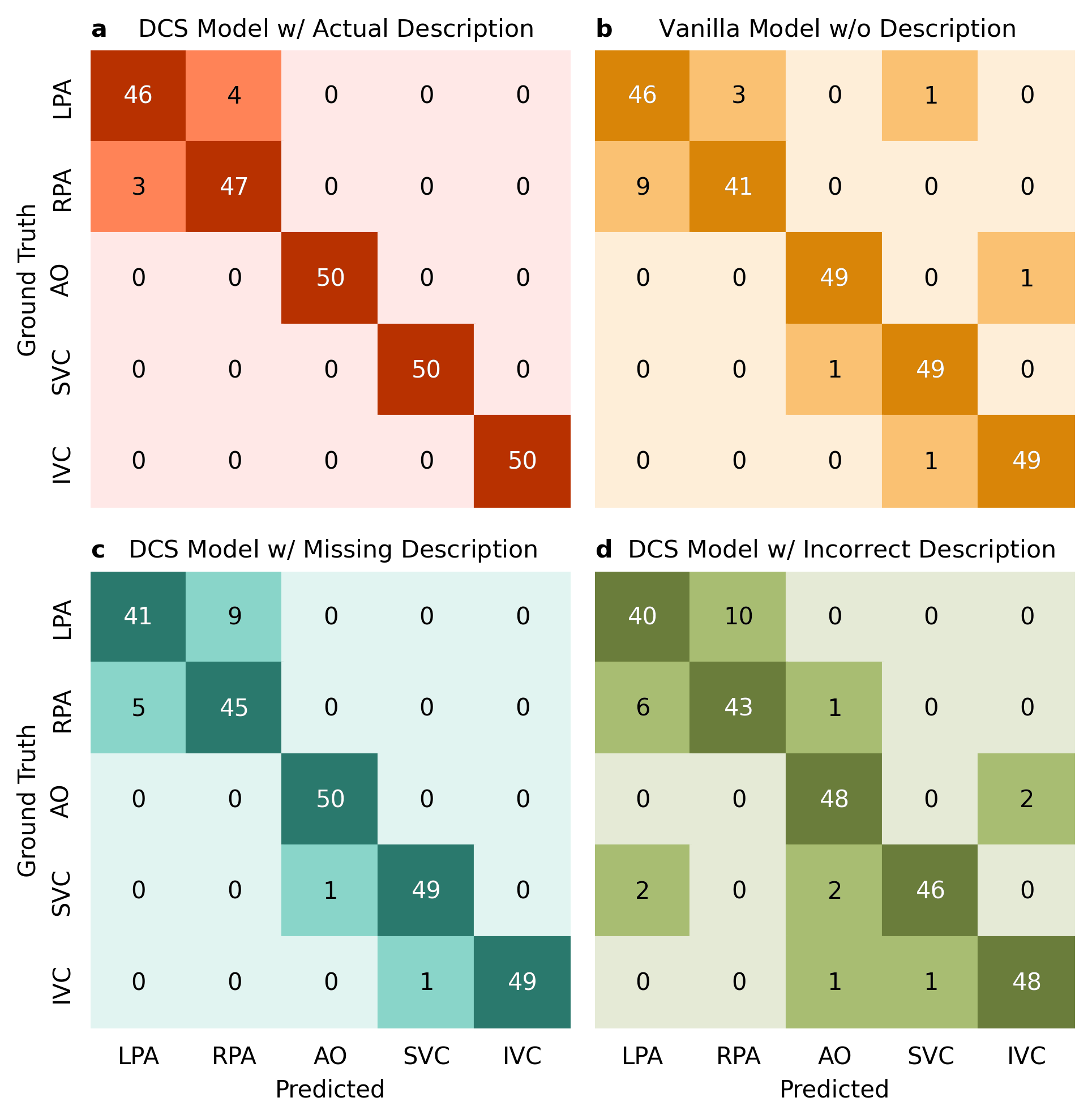


***Supplementary Figure 1. Confusion matrices showing classification accuracy of the Deep Classification + Segmentation (DCS) model on an unseen test set of 50 series.*** *(A) The DCS* *model* *using the actual series descriptions extracted from DICOM headers as input. (B) The “Vanilla” DCS model without the series description input layer. (C) The DCS model using artificially missing series descriptions as input. (D) The DCS model with all series descriptions for every vessel artificially set to be incorrect as input. LPA – Left Pulmonary Artery, RPA – Right Pulmonary Artery, AO – Aorta, SVC – Superior Vena Cava, IVC – Inferior Vena Cava.*


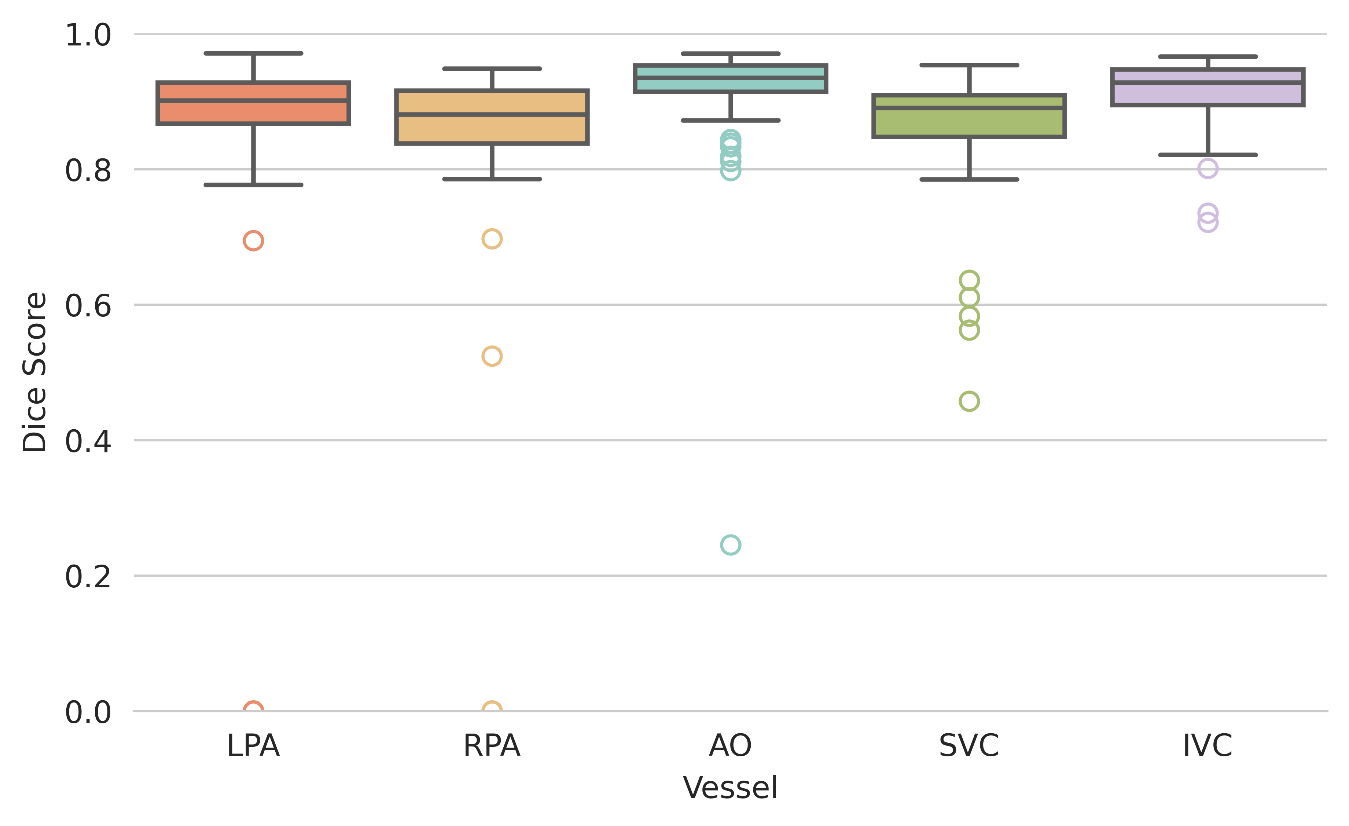
Supplementary Figure 2: Dice score achieved by DCS by distribution by vessel. Box plot showing Dice scores for each vessel across the 50 ground truth test cases.


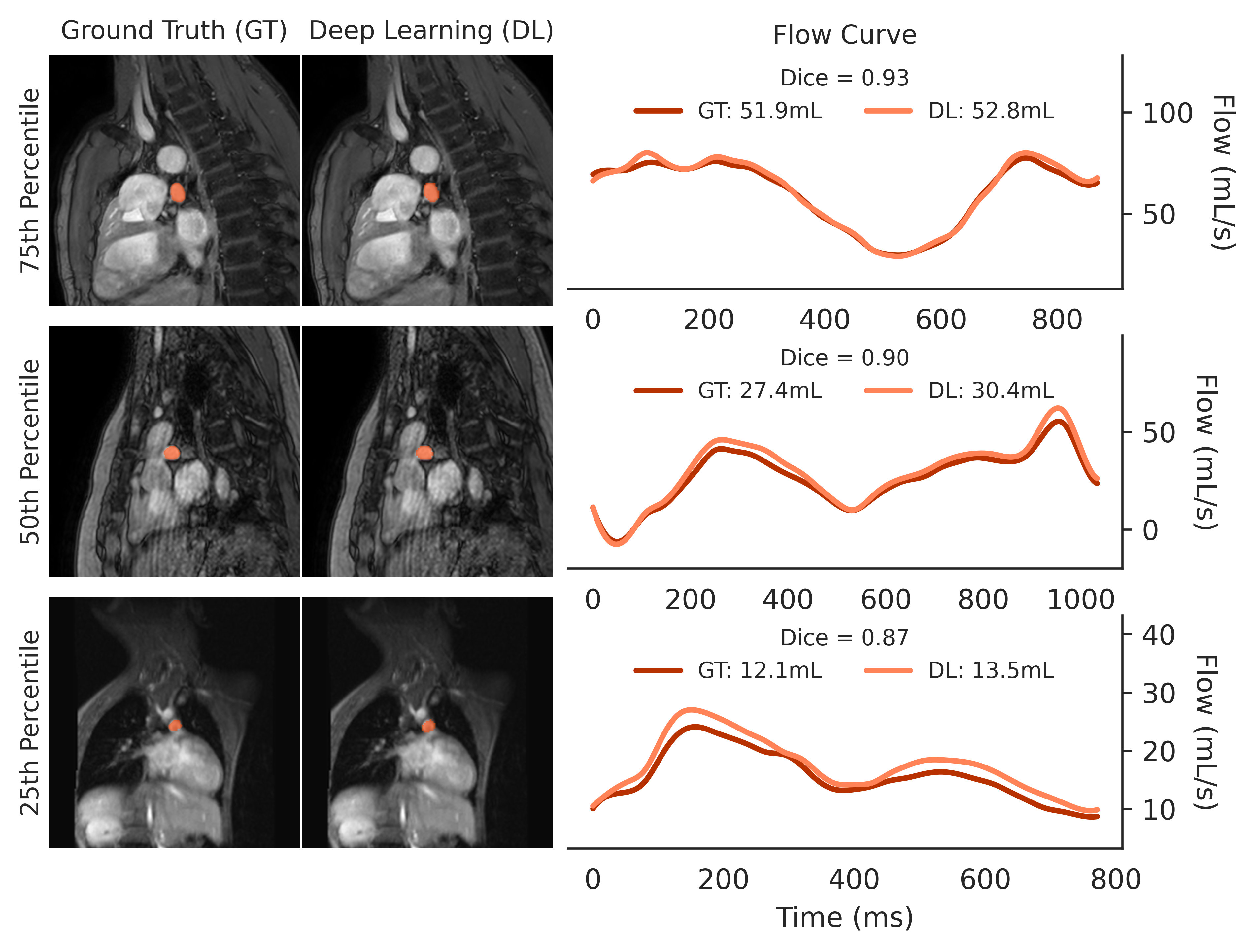


Supplementary Figure 3. Example segmentations of the left pulmonary artery. Ground truth and predictions are shown for test cases at the 25th, 50th, and 75th percentiles of Dice. Only the first frame with its segmentation is displayed, along with the corresponding flow curves and net forward volumes.


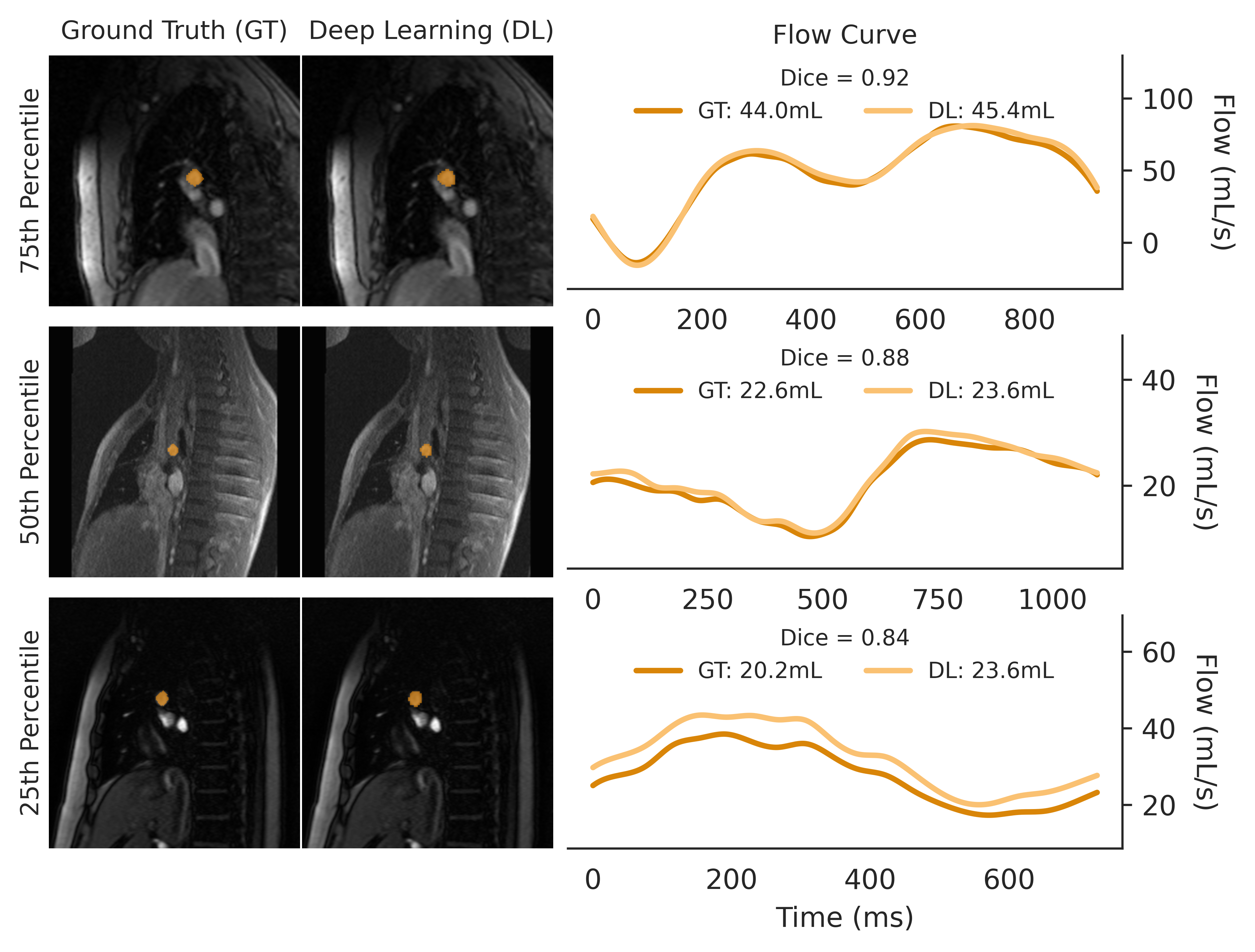
***Supplementary Figure 4. Example segmentations of the right pulmonary artery.*** *Ground truth and predictions are shown for test cases at the 25th, 50th, and 75th percentiles of Dice. Only the first frame with its segmentation is displayed, along with the corresponding flow curves and net forward volumes.*


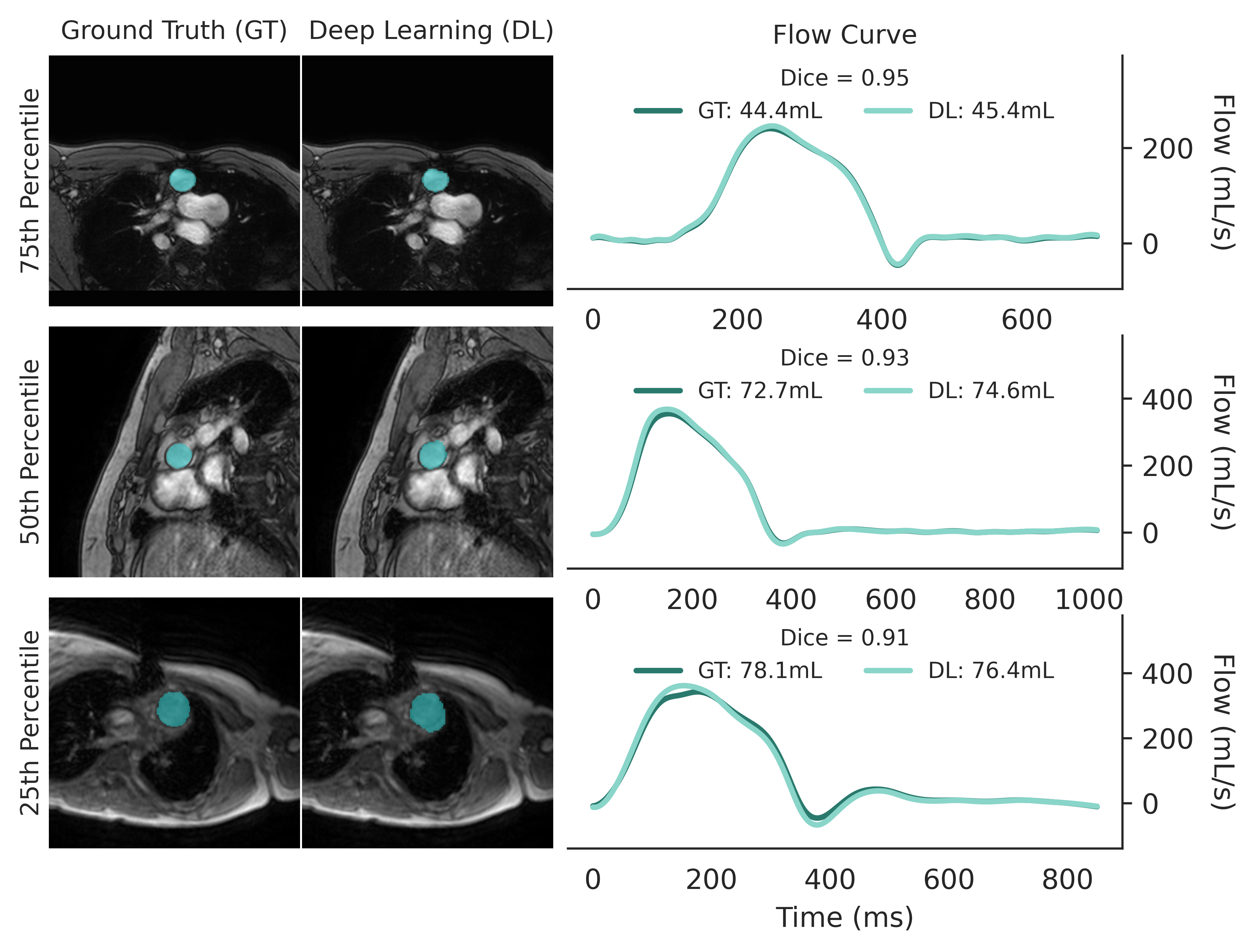
***Supplementary Figure 5. Example segmentations of the aorta.*** *Ground truth and predictions are shown for test cases at the 25th, 50th, and 75th percentiles of Dice. Only the first frame with its segmentation is displayed, along with the corresponding flow curves and net forward volumes.*


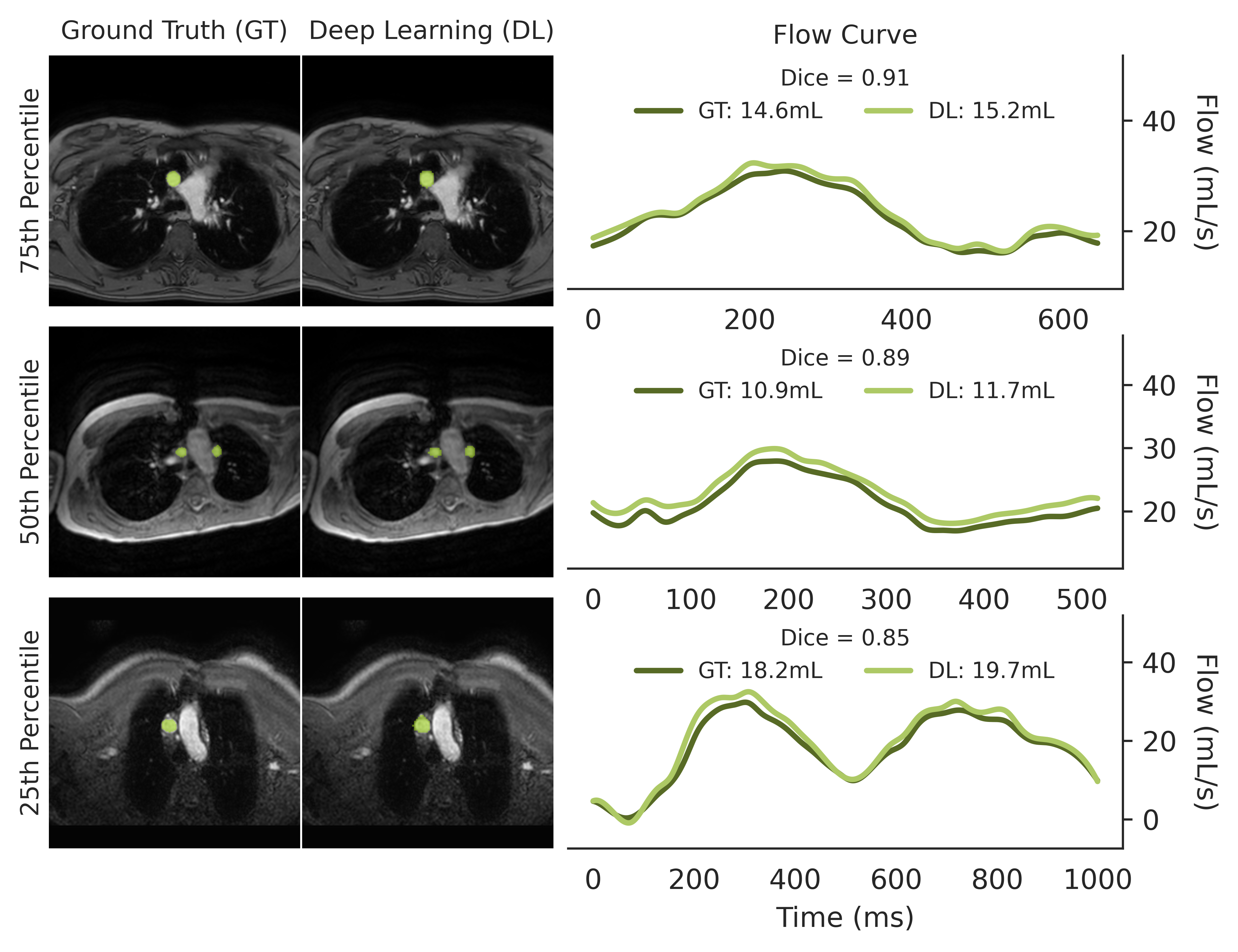
***Supplementary Figure 6. Example segmentations of the superior vena cava.*** *Ground truth and predictions are shown for test cases at the 25th, 50th, and 75th percentiles of Dice. Only the first frame with its segmentation is displayed, along with the corresponding flow curves and net forward volumes.*


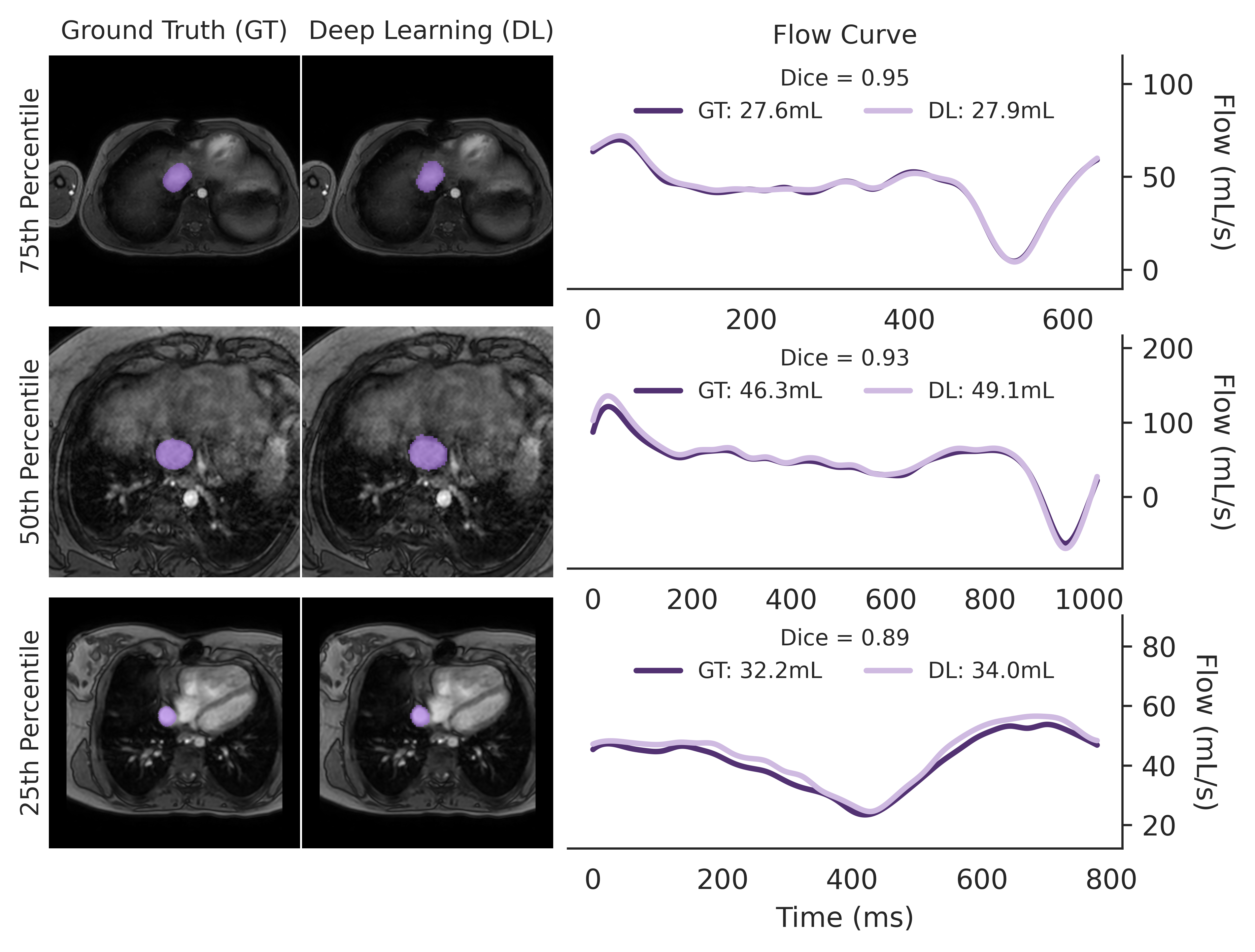
***Supplementary Figure 7. Example segmentations of the inferior vena cava.*** *Ground truth and predictions are shown for test cases at the 25th, 50th, and 75th percentiles of Dice. Only the first frame with its segmentation is displayed, along with the corresponding flow curves and net forward volumes.*


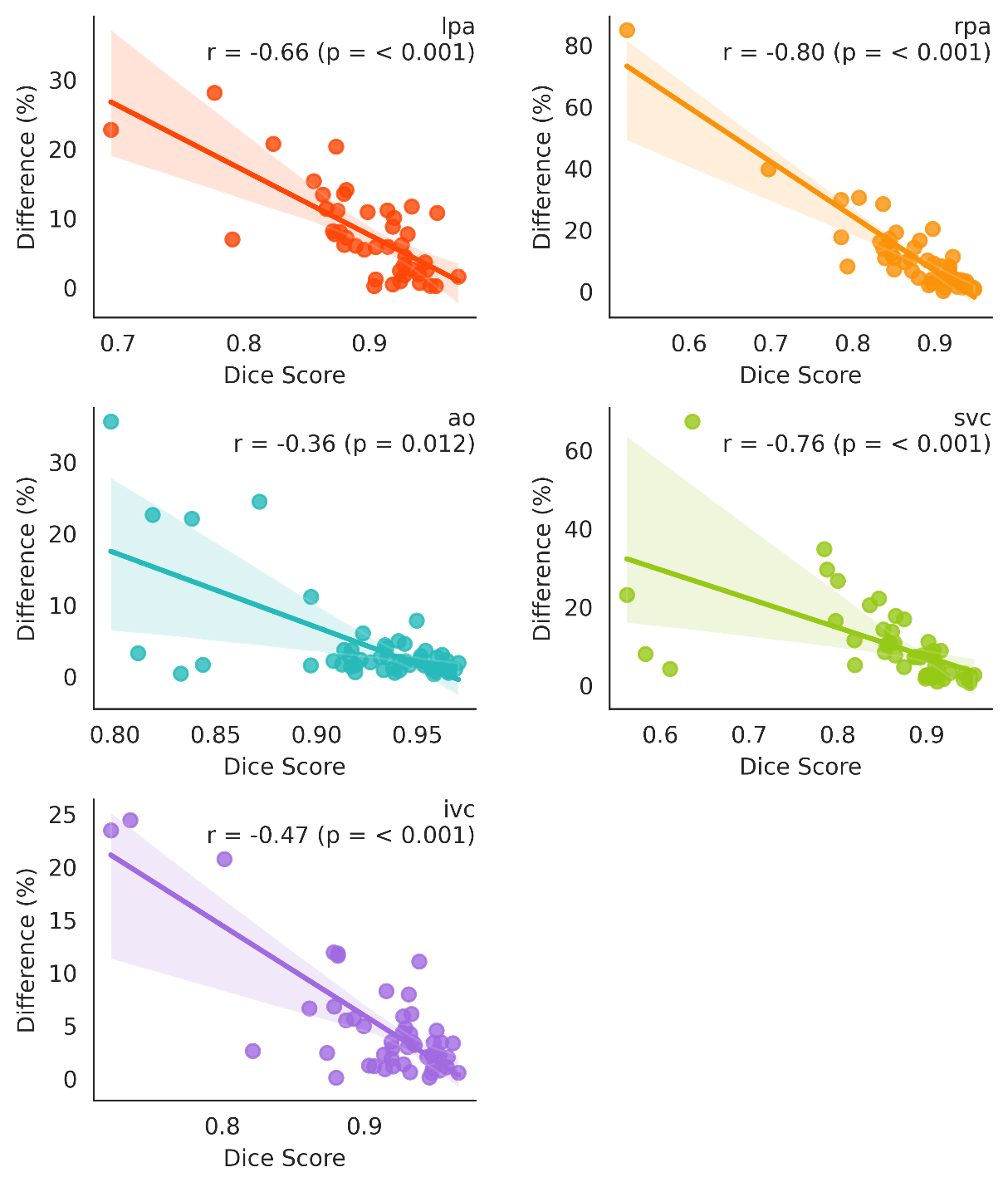


Supplementary Figure 8. Correlation plot between dice score and difference in net forward volume derived from DL and manual segmentations as a percentage for the 50 test set. LPA – Left Pulmonary Artery, RPA – Right Pulmonary Artery, AO – Aorta, SVC – Superior Vena Cava, IVC – Inferior Vena Cava.


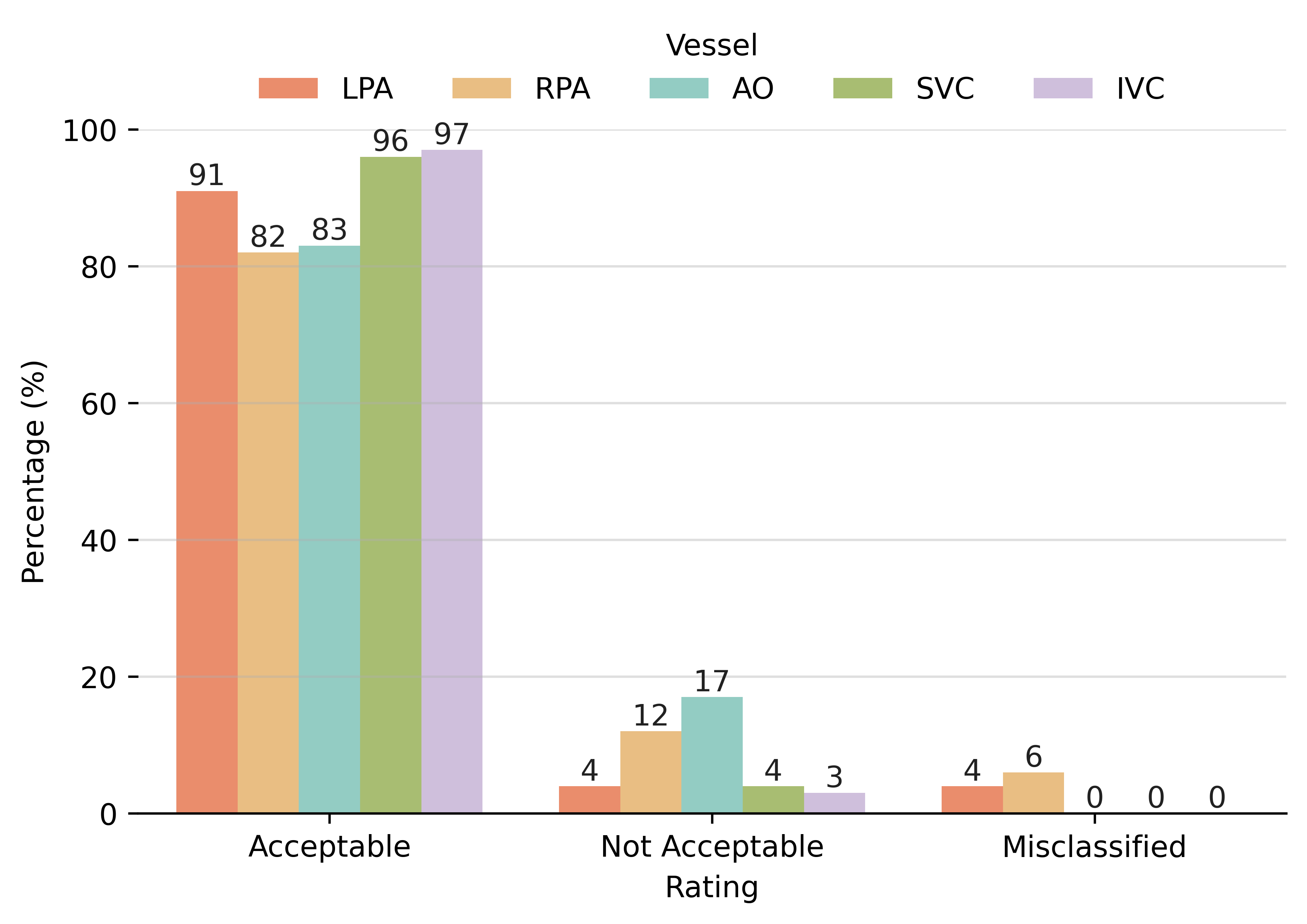
***Supplementary Figure 9. Bar plot of the qualitative review of five vessel segmentations in the pipeline validation dataset.*** *N = 2902 exams, 14510 individual 2D+time segmentations.*


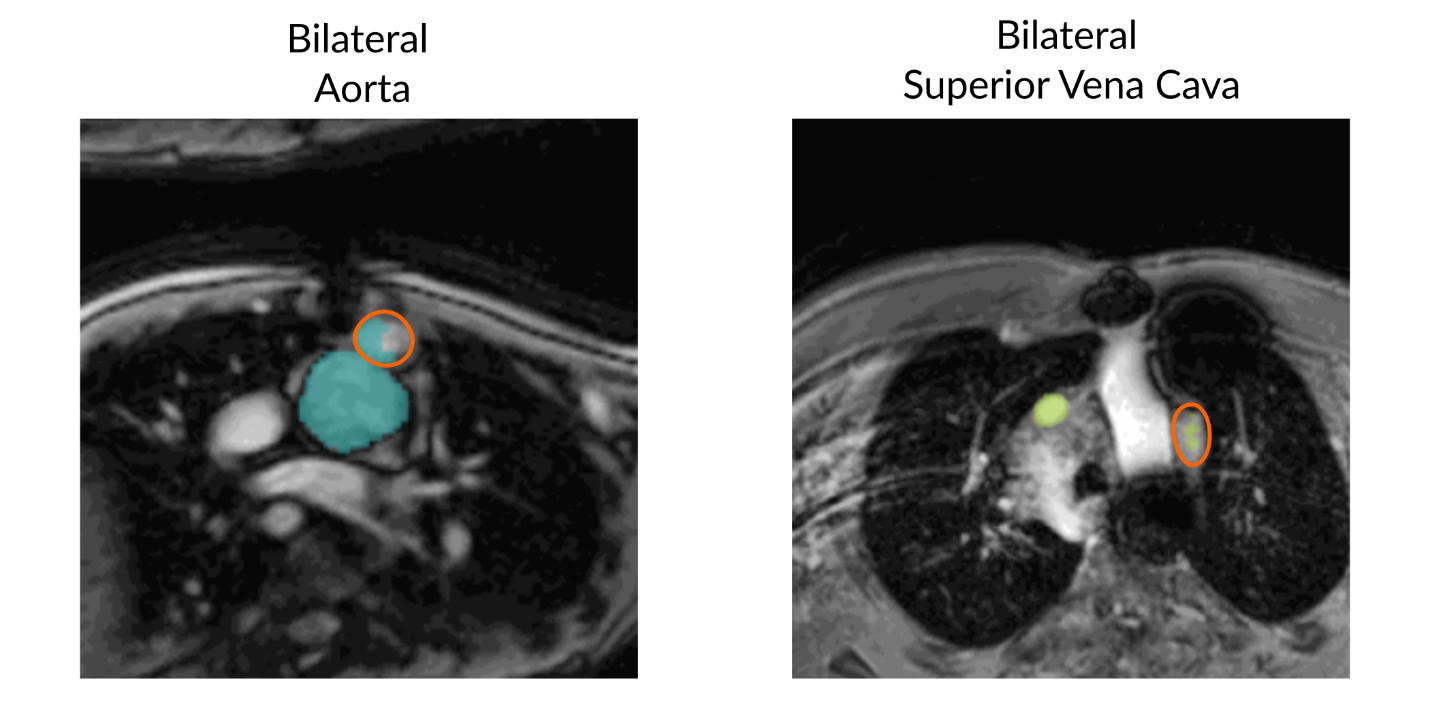
Supplementary Figure 10. Examples of failed segmentations in phase-contrast imaging planes: left) bilateral aorta, right) bilateral superior vena cava. The red circle shows where the segmentation has failed.


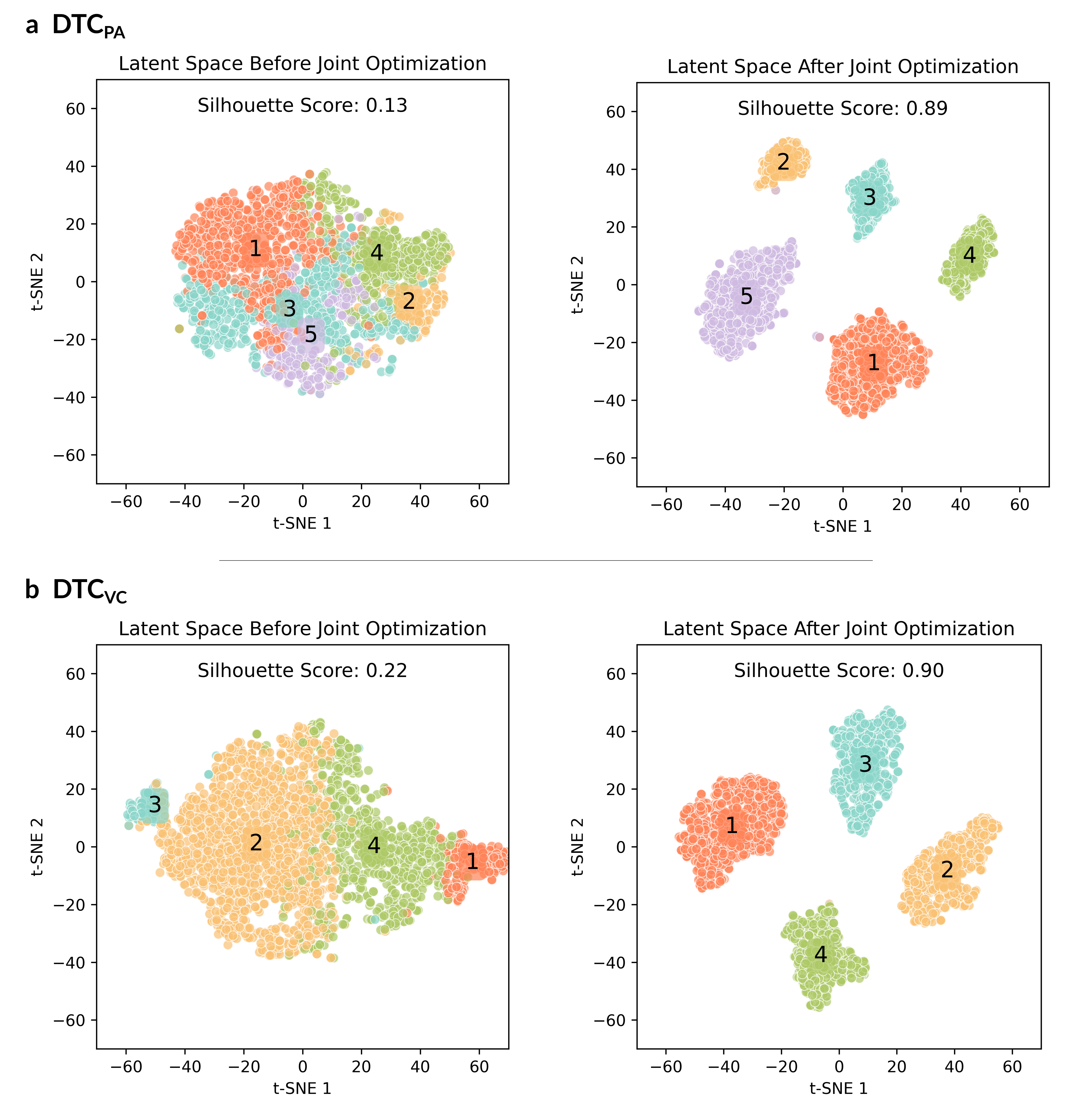
***Supplementary Figure 11. Latent space clusters before and after joint optimization.*** *t-distributed Stochastic Neighbor Embedding (t-SNE) plots of the latent space shown before and after joint optimization, for the a) DTC_PA_ and b) DTC_VC_ models.*


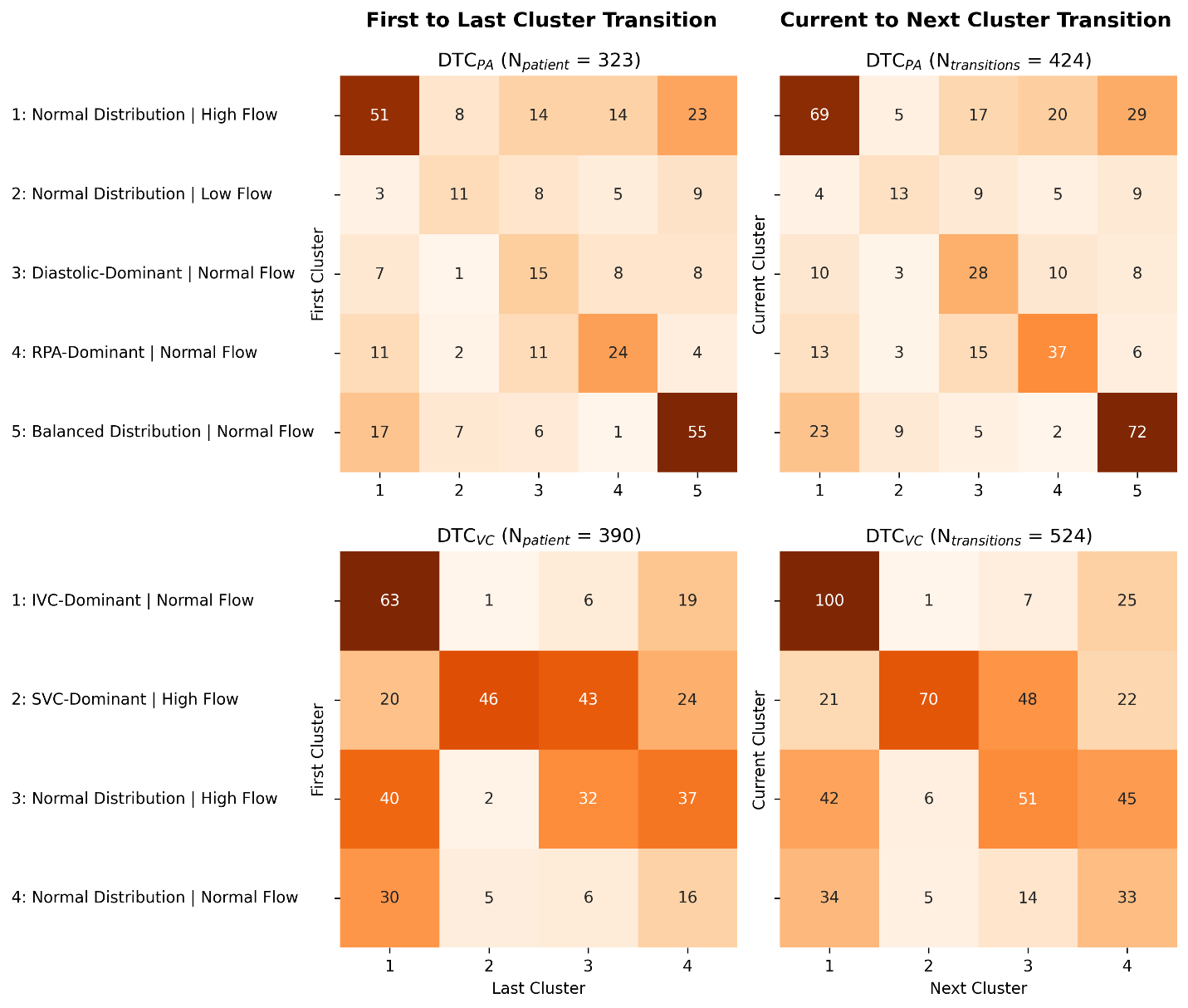
Supplementary Figure 12. Heatmaps showing the cluster transitions in the DTC_PA_ and DTC_VC_ models for patients with multiple scans. The left column displays the cluster transitions from the first to last scan of each patient, while the right column shows consecutive cluster transitions from each scan to the patient’s next scan. The plot visualises clusters stability (diagnonal values) and transitions between clusters (off-diagonal values).


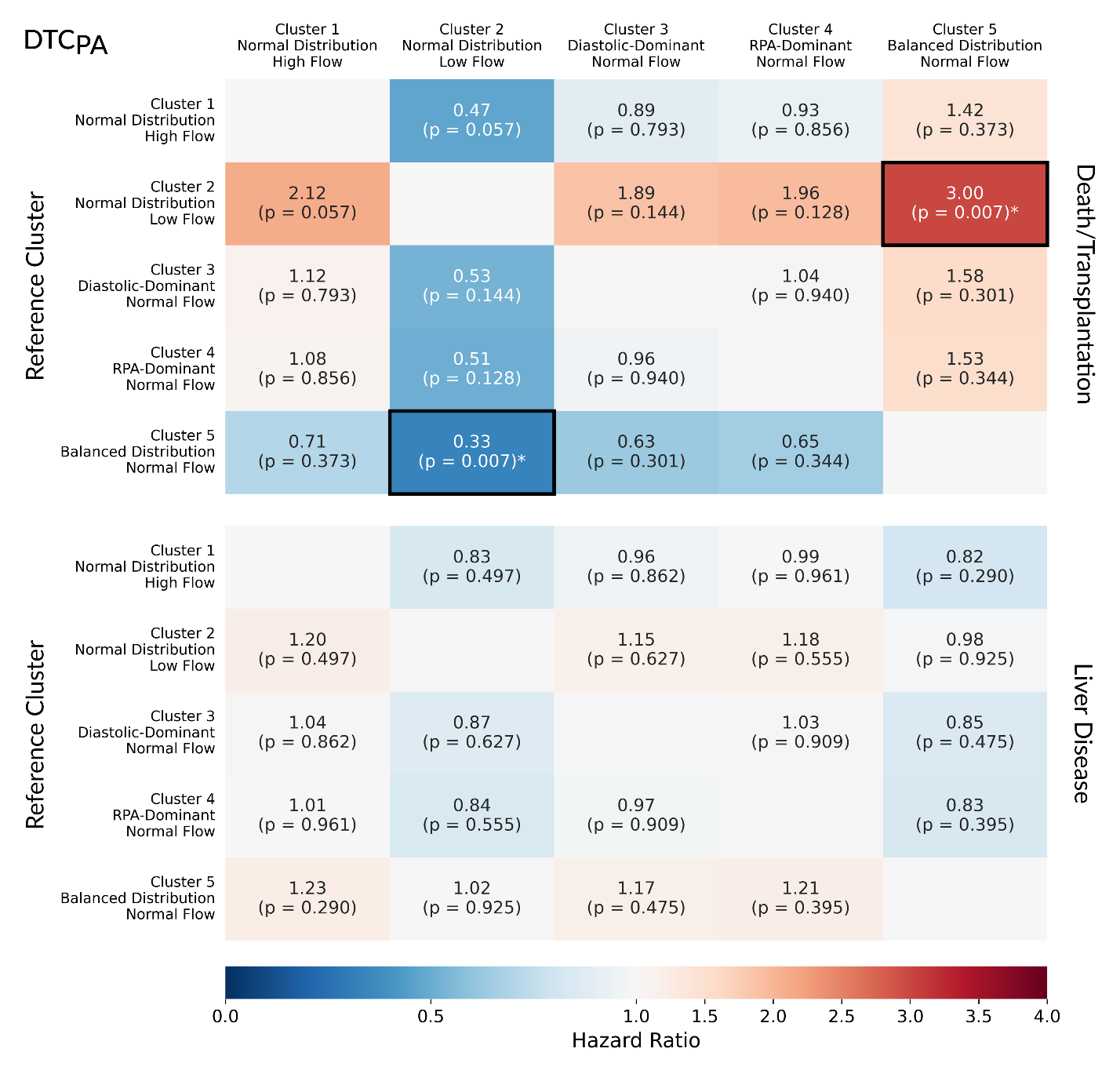


***Supplementary Figure 13. Heatmaps of hazard ratios for death/transplantation and liver disease outcomes based on time-varying Cox regression survival analysis across the five clusters identified by DTC_PA_.*** *Models were adjusted for age, sex, indexed aortic flow rate, and ejection fraction. For each comparison, the cluster on the y-axis is the reference group, and hazard ratios indicate the risk of each x-axis cluster relative to that reference. * denotes significant differences survival between compared clusters.*


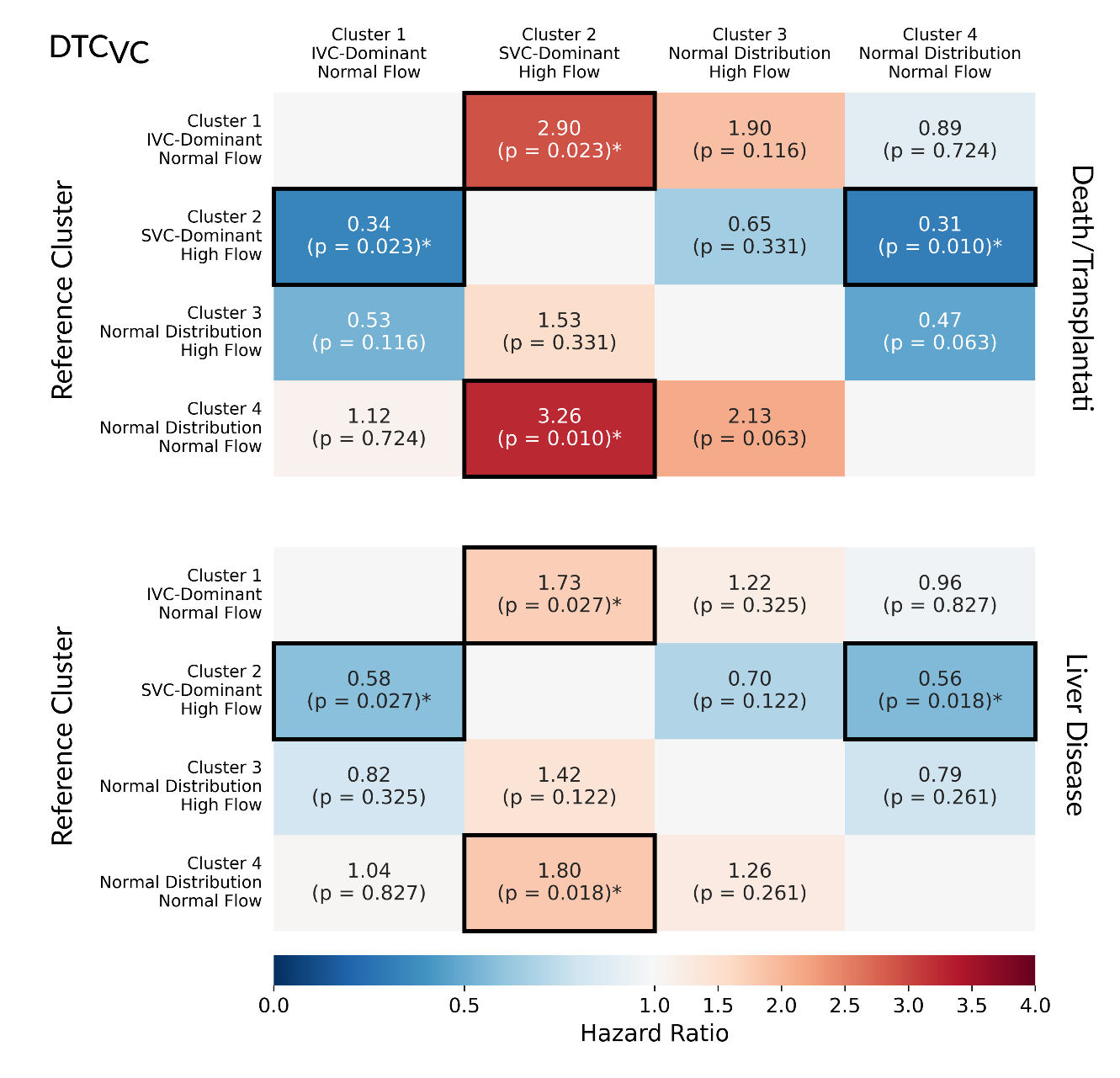


***Supplementary Figure 14. Heatmaps of hazard ratios for death/transplantation and liver disease outcomes based on time-varying Cox regression survival analysis across the four clusters identified by DTC_VC_.*** *Models were adjusted for age, sex, indexed aortic flow rate, and ejection fraction. For each comparison, the cluster on the y-axis is the reference group, and hazard ratios indicate the risk of each x-axis cluster relative to that reference. * denotes significant differences survival between compared clusters.*


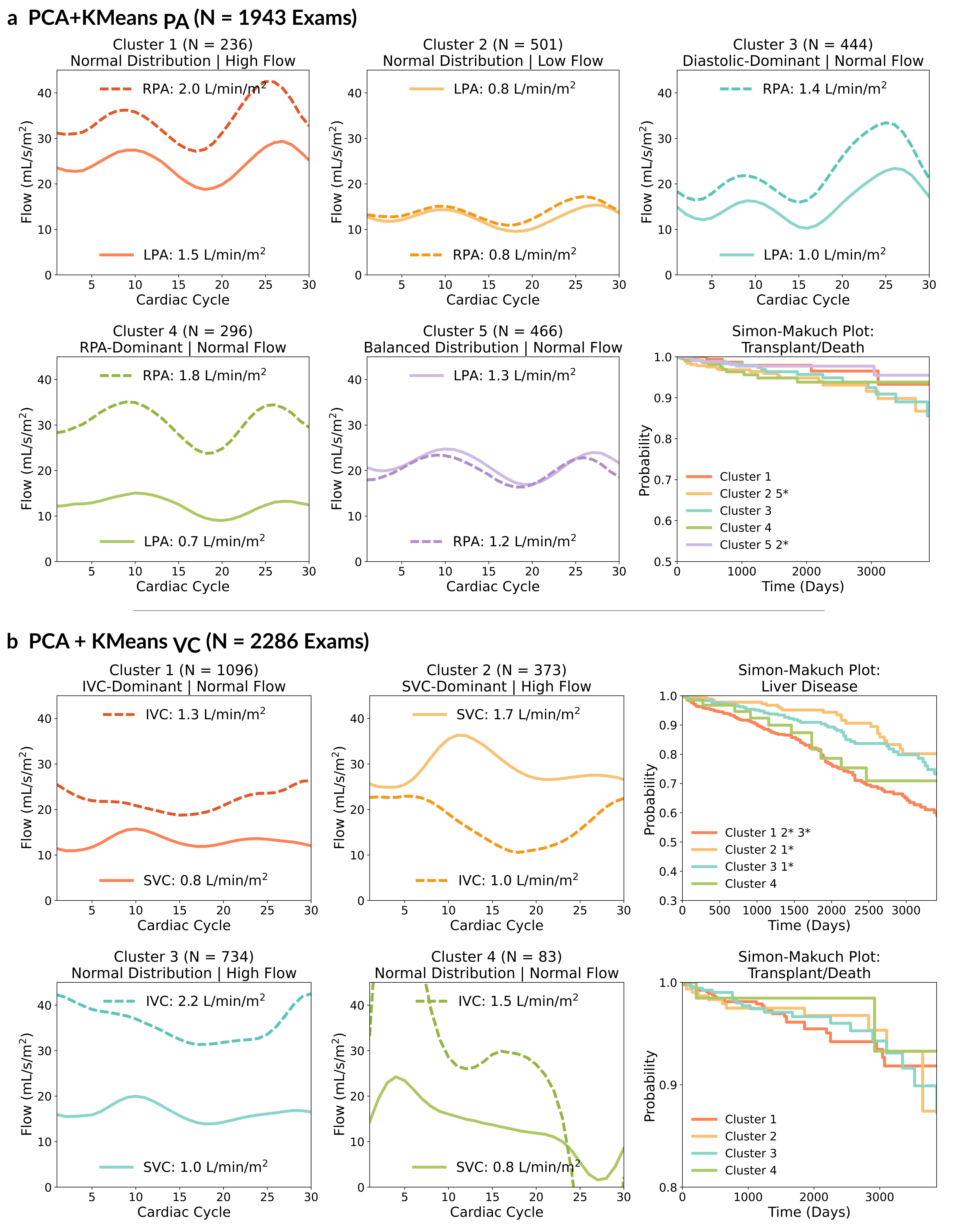


Supplementary Figure 15. Median flow curves for each cluster and the mean flow for each vessel obtained from k-means clustering on PCA-reduced flow curve components, shown for comparison with the DTC model. *The shaded regions indicate the confidence interval. LPA = Left Pulmonary Artery, RPA = Right Pulmonary Artery, SVC = Superior Vena Cava, IVC = Inferior Vena Cava. * Marks significant cluster differences according to time-varying Cox Regression.*


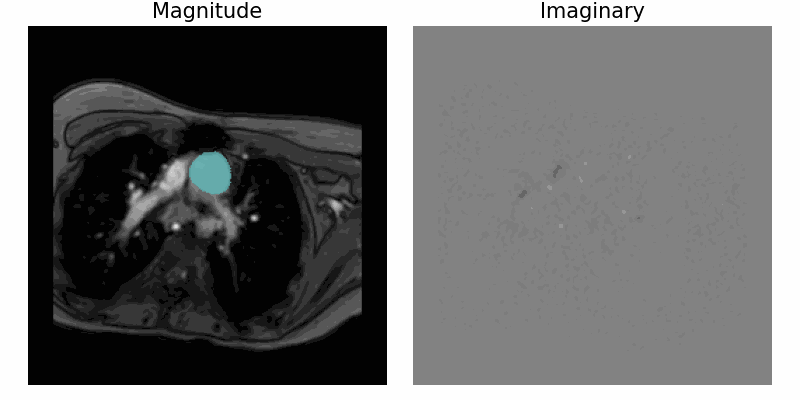


Supplementary Movie 1. Example input pair of magnitude and imaginary images used in the DCS model. The imaginary component highlights flowing blood with high signal intensity, while static tissue exhibits near-zero signal.

### Supplementary Section 1: Pipeline Overview

The first step was to identify all phase images using DICOM header information (non-zero velocity-encoding value (VENC) with a minimum of 20 time points). The paired magnitude images were then identified by matching orientation (Image Orientation Patient Attribute), position (Image Position Patient Attribute), and ensuring that the image creation times (Instance Creation Time Attribute) were within five minutes. This approach was robust across different scanner vendors that store phase-contrast images with different protocols. All 2D+time image blocks were subsequently preprocessed and inputted in the DCS model as described above.

It should be noted that a patient exam may contain more PCMR series than the five vessel planes on which the model was trained. The DCS model will attempt to classify these series into one of our five categories, potentially resulting in misclassification (e.g. left pulmonary vein misclassified as the left pulmonary artery or a scan plane). If multiple series were classified as the same vessel, and only one has a series description that is consistent with the model classification then that one is chosen (e.g., if the classification is 'LPA' and the description includes 'LPA'; see Supplementary Table 8 – Reference Data Dictionary). If this is not the case, we pick the one with the highest probabilities from the classification-guided module.

Once all vessels had been identified, flow curves and net forward volumes were calculated as described above and for quality assurance GIFs of the segmented images were generated. To evaluate pipeline robustness, a single researcher reviewed each exam, categorizing vessel segmentation as ‘acceptable’, ‘not acceptable’, or categorizing the vessel as ‘misclassified’. To be rated as acceptable, segmentation masks had to accurately cover the vessel of interest in all frames without missing areas or over segmentation. Additionally, exams were evaluated for image quality, the presence of bilateral aortas and SVCs, and situs type for each exam.
